# Improving the detection of sleep slow oscillations in electroencephalographic data

**DOI:** 10.3389/fninf.2024.1338886

**Published:** 2024-02-05

**Authors:** Cristiana Dimulescu, Leonhard Donle, Caglar Cakan, Thomas Goerttler, Lilia Khakimova, Julia Ladenbauer, Agnes Flöel, Klaus Obermayer

**Affiliations:** ^1^Department of Software Engineering and Theoretical Computer Science, Technical University Berlin, Berlin, Germany; ^2^Bernstein Center for Computational Neuroscience Berlin, Berlin, Germany; ^3^Department of Neurology, University Medicine, Greifswald, Germany; ^4^German Center for Neurodegenerative Diseases, Greifswald, Germany

**Keywords:** electroencephalography, sleep, slow oscillations, event detection, deep learning

## Abstract

**Study objectives:**

We aimed to build a tool which facilitates manual labeling of sleep slow oscillations (SOs) and evaluate the performance of traditional sleep SO detection algorithms on such a manually labeled data set. We sought to develop improved methods for SO detection.

**Method:**

SOs in polysomnographic recordings acquired during nap time from ten older adults were manually labeled using a custom built graphical user interface tool. Three automatic SO detection algorithms previously used in the literature were evaluated on this data set. Additional machine learning and deep learning algorithms were trained on the manually labeled data set.

**Results:**

Our custom built tool significantly decreased the time needed for manual labeling, allowing us to manually inspect 96,277 potential SO events. The three automatic SO detection algorithms showed relatively low accuracy (max. 61.08%), but results were qualitatively similar, with SO density and amplitude increasing with sleep depth. The machine learning and deep learning algorithms showed higher accuracy (best: 99.20%) while maintaining a low prediction time.

**Conclusions:**

Accurate detection of SO events is important for investigating their role in memory consolidation. In this context, our tool and proposed methods can provide significant help in identifying these events.

## 1 Introduction

During sleep, the brain displays a set of characteristic oscillatory rhythms, one prominent rhythm being the slow wave sleep (SWS) regime, defined by the presence of slow oscillations (SOs) (Rasch and Born, 2013). While the term “SO” has received some criticism (Timofeev et al., 2020), it is generally used in the literature to refer to neocortical large-amplitude events with a low frequency observed most prominently over fronto-central regions, nevertheless, important differences exist in this definition across studies. In particular, criteria for SO amplitude can vary. Massimini et al. (2004) uses an absolute amplitude threshold of 140 μV, Muehlroth and Werkle-Bergner (2020) uses 70 μV, whereas others determine this value dynamically for each individual participant (Mölle et al., 2009; Ladenbauer et al., 2017). Additionally, some studies distinguish between delta (~1 to 4 Hz) and SO (0.1 to ~1 Hz) (Ladenbauer et al., 2017) frequency, whereas others combine the two (Massimini et al., 2004). However, the dichotomy between the two is supported by differences in the respective cellular correlates of these oscillations (Amzica and Steriade, 1998), differences in origin (Amzica and Steriade, 1998; Timofeev et al., 2000), and in physiological and functional correlates (e.g., differential homeostatic regulation: Achermann and Borbély, 1997; Bersagliere and Achermann, 2010; and memory function and in the context of cognitive pathology in aging: Mander et al., 2015). Despite these variations in definitions, from a functional perspective, SOs are important for memory consolidation. According to the “active system consolidation hypothesis,” SOs play a crucial role in this process, as they help mediating the transfer of hippocampal-dependent memory representations to the neocortical long-term storage (Rasch and Born, 2013).

Studies investigating the effect of auditory stimulation during sleep have shown that the number of SOs increases after stimulation and that this correlates with improved declarative memory performance (Ngo et al., 2013). Additionally, both the SO amplitude and the duration of the SO up-state increase after a learning task and this increase correlates with task performance after sleep (Heib et al., 2013). Finally, the synchronization between SOs and thalamocortical sleep spindles [10–15 Hz waxing and waning activity (Rasch and Born, 2013)] appears to be crucial for memory consolidation and transcranial current stimulation (tCS) protocols which boost this coupling lead to better memory performance in a variety of tasks (Ladenbauer et al., 2017; Mikutta et al., 2019; Muehlroth et al., 2019).

A fundamental methodological assumption in these studies, however, is the fact that SOs are reliably and comparably identified across studies. SO detection often relies on automatic detection methods applied to the electroencephalography (EEG) signal. Typically, such algorithms are applied to EEG signals bandpass-filtered within a variable low-frequency range and SOs are selected based on a series of amplitude and peak detection criteria (Massimini et al., 2004; Mölle et al., 2009; Ladenbauer et al., 2017; Muehlroth et al., 2019). These criteria can be applied in the form of either fixed or individually-adjusted, i.e., relative, thresholds. Given that both the bandpass filter frequency ranges and the threshold values can vary across studies, it is unclear to what extent results from different studies can be compared to each other.

Comparison of results across studies is further complicated when taking into account age differences. Numerous studies show that older adults display reduced SO numbers, amplitudes, and frequencies (Dijk et al., 2000; Edwards et al., 2010). According to Muehlroth and Werkle-Bergner (2020), both fixed and relative threshold algorithms lead to similar results for young adults, but they show marked differences for older adults. In particular, as SO amplitudes are reduced in older adults, fixed threshold algorithms tend to miss more SO events compared to the relative threshold ones (Muehlroth and Werkle-Bergner, 2020).

Consequently, while automatic SO detection algorithms offer an immense advantage in terms of data labeling speed, they carry several disadvantages, including difficulties in comparing results across studies, as well as an apparent lack of reliable detection across age groups. Thus, comparison of results from manually expert-labeled events with results from different automatic SO detection algorithms might help address these challenges. Nevertheless, to the authors' best knowledge, such a manually labeled validation data set is currently not publicly available.

In this work, we address the issue of validation data by first implementing a tool which facilitates the manual labeling of SO events. Subsequently, we use this tool in order to produce a manually labeled validation data set based on the EEG recordings of ten older adults and compare the performance of three automatic SO detection algorithms against the validation data. Additionally, we compare common SO features, such as density and amplitude, and observe that the three automatic SO detection algorithms lead to diverging results. Finally, we use this benchmark data set to test three approaches using machine learning and deep learning methods, which can achieve better results for SO detection compared to the traditional ones. Based on our results, we propose a recommended workflow for optimal SO labeling and detection for future sleep EEG studies.

## 2 Method

### 2.1 Data

#### 2.1.1 Participants

Ten older adults (five females, age range = 50–77 years, mean age = 60.9 years) gave their written informed consent for participating in the current sleep study conducted at the Universitätsmedizin Greifswald. Daytime sleep EEG recordings were acquired. The study was approved by the local ethics committee at the Universitätsmedizin Greifswald and was in accordance with the Declaration of Helsinki. All participants were reimbursed for their participation.

#### 2.1.2 Acquisition

EEG data were recorded during 90-min afternoon naps as part of a larger study in which the effect of slow oscillatory transcranial direct current stimulation (so-tDCS) (Ladenbauer et al., 2021) was investigated. For the purposes of the current study, we selected baseline recordings from seven participants, together with so-tDCS recordings from two participants and one sham recording from one participant. The data were acquired from scalp sites using Ag/AgCl active ring electrodes incorporated into an EEG cap according to the extended 10–20 international EEG system, using the Brain Vision Recorder software at a sampling rate of 500 Hz and were referenced to an electrode attached to the nose. 28 scalp electrodes (FP1, FP2, AFz, F3, F4, F7, Fz, F8, FC5, FC1, FC2, FC6, C3, Cz, C4, T7, T8, CP5, CP1, CP2, CP6, P7, P3, Pz, P4, P8, O1, O2) were used in the baseline recordings and 26 in the so-tDCS and sham recordings (F3 and F4 were replaced by stimulation electrodes in this case). Simultaneously, following the standard sleep monitoring protocol, chin electromyography (EMG) and electrooculography (EOG) data were recorded.

#### 2.1.3 Sleep stage classification

One medical expert used the Schlafaus software (Steffen Gais, Lübeck, Germany) to manually perform the sleep stage classification on the raw data downsampled to 250 Hz, based on the standard criteria outlined in Rechtschaffen (1968). For this purpose, the expert used 30 s epochs, classifying each as belonging to one of seven stages: wakefulness (Wa), non-REM sleep stage 1 (N1), 2 (N2), 3 (N3), or 4 (N4), REM sleep or movement artifact. All ten participants reached sleep stages N1 and N2, while seven also reached sleep stage N3. No participant displayed N4 activity during the afternoon nap. Eight participants also had periods of awake time.

#### 2.1.4 Preprocessing

We preprocessed the EEG data using custom scripts implemented in the FieldTrip toolbox (Oostenveld et al., 2011). The preprocessing pipeline was the same as described in Cakan et al. (2022). In brief, the first step involved identifying artifactual independent components (ICs) in the data to be removed in the second preprocessing step. For that, a 1–100 Hz bandpass finite impulse response filter and a 50 Hz bandstop filter with a bandwidth of 4 Hz were applied to the data. Afterwards, we manually removed gross noise portions affecting all EEG channels, excluded the EMG channels, and conducted an independent component analysis (ICA) with 30 ICs using the *runica* algorithm (Makeig et al., 1997). These ICs were used to identify and remove artifacts from the data. In particular, we used scalp topographies (Jung et al., 1997, 2000) and power spectra (Criswell, 2010) to identify and remove ICs corresponding to muscle artifacts, heart beat, and eye movements.

Starting again from the raw data for our main analyses, we applied a finite impulse response bandpass filter between 0.1 and 100 Hz, coupled with a 50 Hz bandstop filter with a bandwidth of 4 Hz to the data, then removed all artifactual ICs identified in the previous step, as well as the EOG channels, and segmented the data into 10 s epochs. In order to detect any remaining artifact-contaminated channels, as well as artifact-contaminated epochs, we used a two-step procedure. The first step involved the detection of outliers exceeding four times the standard deviation of the kurtosis of the power distribution in the low- (0.1–2 Hz) and high-frequency (30–100 Hz) bands. The second step made use of the FASTER algorithm (Nolan et al., 2010). Channels containing artifacts were interpolated using spherical splines (Perrin et al., 1989), and epochs were removed. Finally, all remaining 10 s epochs were visually inspected and those still containing artifacts were removed.

### 2.2 Semi-automatic SO labeling tool

We implemented a custom tool for manually labeling SO events in EEG data available in Donle et al. (2022). As SO events are relatively sparse in EEG recordings, the tool speeds up the manual labeling process by filtering out most data and only displaying potential SO events. Potential SO events are defined by two consecutive positive-to-negative zero-crossings with a user-defined duration. A description of the interface used can be found in Section 3.1.

Additionally, we developed a semi-automatic pre-filtering procedure for further reducing the number of events to be manually inspected, thus decreasing the time needed for this task. The pre-filtering and manual labeling procedures are described in Sections 2.2.1 and 2.2.2.

#### 2.2.1 Pre-filtering

While the SO labeling tool filters out portions of the data where no event with SO characteristics exists, the number of events to be manually inspected remains relatively large, with up to ~60,000 events per EEG recording. Most of these events are false positives. To further reduce the time needed for manually labeling our EEG recordings, we developed a pre-filtering procedure to discard as many false positive events as possible, while still retaining a large number of true positive events.

**Figure 1:**
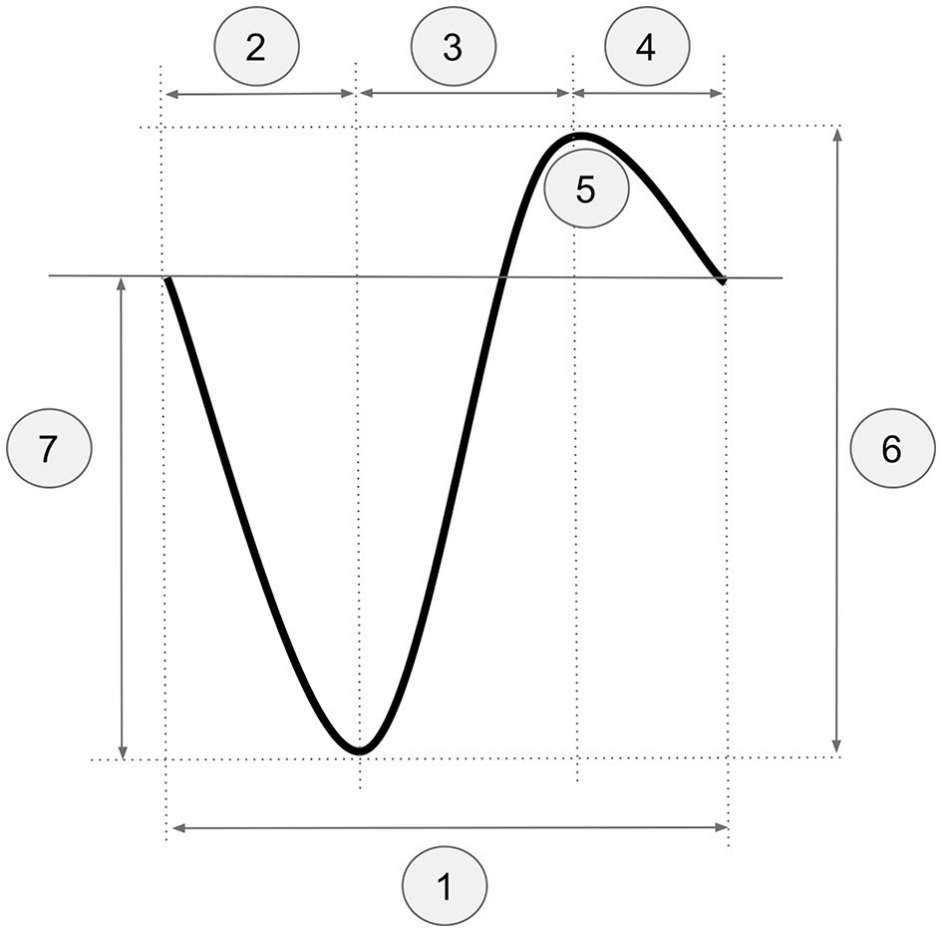
Criteria used for the pre-filtering procedure exemplified on schematic SO event: (1) total SO duration, (2) duration from the first positive-to-negative zero-crossing to the negative peak, (3) duration from the negative to the positive SO peaks, (4) duration from the positive peak to the second positive-to-negative zero-crossing, (5) number of positive wave peaks, (6) scaling factor for negative-to-positive peak-to-peak amplitude, (7) scaling factor for negative peak voltage.

Firstly, for each participant, one channel was randomly selected, and all events with a duration of 0.8–3.5 s between two consecutive positive-to-negative zero-crossings were identified. The random channel selection was motivated by wanting to ensure that the results of the procedure were independent of channel selection. One rater then manually labeled the events as SO or non-SO. Secondly, for all selected channels, we calculated the mean and standard deviation of the following five parameters (see Figure 1): (1) total SO duration, (2) duration from the first positive-to-negative zero-crossing to the negative peak, (3) duration from the negative to the positive SO peaks, (4) duration from the positive peak to the second positive-to-negative zero-crossing, and (5) number of positive wave peaks. Additionally, the minimum and average negative-to-positive peak-to-peak amplitude and the minimum and average negative peak voltage were calculated. These values were used to define the scaling factors for the negative-to-positive peak-to-peak amplitude and the negative peak voltage, respectively (see below).

As the optimal values for SO selection for these parameters were not known *a priori*, we examined a range of potential values based on the data features calculated above and used the true and false positive rates as evaluation metrics. For each of the first five features listed above, we examined nine equally spaced values in the interval defined by the mean ± two standard deviations. For every value, we used the manually labeled events to calculate the true positive rate (TPR) for that respective channel as follows:


(1) $$\begin{array}{l} TPR=\frac{TP}{TP+FN}, \end{array}$$

and the false positive rate (FPR) as:


(2) $$\begin{array}{l} FPR=\frac{FP}{FP+TN}, \end{array}$$

where *TP* = number of true positives, *FN* = number of false negatives, *FP* = number of false positives, and *TN* = number of true negatives in that channel. Finally, starting from the maximum criterion value (i.e., mean + 2 SDs; for the number of positive peaks, the values are first rounded to the nearest integer) for each parameter, we calculated the difference in both TPR (Equation 1) and FPR (Equation 2) obtained for the current criterion value and the next smaller one. The choice of the maximum value as the starting point as opposed to the minimum was motivated by the fact that we were interested in preserving as many true positive events as possible. Accordingly, if the difference between the two TPRs was larger than the difference between the two FPRs (indicating a larger loss of TPs compared to FPs), we stopped the procedure and set that parameter's value to the current criterion value. Otherwise we repeated the procedure for the next criterion value. This stopping criterion was motivated by the fact that a loss of true positive events is acceptable as long as it is balanced out by a larger reduction of false positives. An example for this procedure is shown in the Supplementary Figure S1, and the chosen values for all criteria for each participant are shown in Supplementary Table S1. As the number of false positives in the data is too large for manual labeling to be feasible, losing some true positives for the benefit of removing a large number of false positives is necessary. To test whether the loss of true positives significantly affects the generalization of the machine learning and deep learning algorithms to out-of-sample events, we also report algorithm performance on the fully labeled single-channel data, where no pre-filtering procedure was applied (see Section 2.7), and from which we excluded the training events.

For the last two parameters, i.e., scaling factor for the negative-to-positive peak-to-peak amplitude and scaling factor for the negative peak voltage, the threshold was defined as the minimum parameter value divided by the average value of that channel. The minimum SO duration was kept fixed at 0.8 s, and all events with a lower duration value were excluded.

The percentage of remaining TPs and FPs for each participant's manually labeled channel after the pre-filtering procedure was applied are outlined in the Supplementary Table S2. While for some participants up to 20% of the true positive events are lost, up to 97% of the false positives are also eliminated. This considerably reduces the workload, as the number of events to be manually inspected decreases from ~60,000 to ~10,000 per recording.

#### 2.2.2 Labeling

Prior to manual labeling, preprocessed data were filtered between 0.1 and 1.25 Hz using a 2^nd^ order Butterworth filter, and positive-to-negative zero-crossings were identified. The parameter values derived in Section 2.2.1 were applied for pre-filtering the data. The data were then labeled by two trained experts (one doctoral candidate and one student assistant, both trained by a postdoctoral researcher in the Department of Neurology at Universitätsmedizin Greifswald) using our labeling tool *Sleepy*, which displays potential SO events and allows labeling them in a sequential manner. We chose to display the events individually in order to eliminate any potential bias toward labeling only global events present in multiple channels and increase the chances of capturing local SO events as well. Large-amplitude events, with a total duration between ~0.8–2 s and overall shape similar to that depicted in Figure 1, were marked as SOs by the experts. The tool is available under https://github.com/caglorithm/sleepy, and a description of the used interface can be found in Section 3.1.

#### 2.2.3 Performance of the semi-automatic SO labeling tool

To determine the degree of consensus between the medical experts and thus to assess whether SOs can be reliably identified by independent observers, we calculated the inter-rater agreement across all participants included in the dataset as follows:


(3) $$\begin{array}{l} IR_{agr}=\frac{SO_{both}+nonSO_{both}}{events_{total}}, \end{array}$$

where *SO*_*both*_ represents the number of events marked as an SO by both raters, *nonSO*_*both*_ the number of events marked as a non-SO by both raters, and *events*_*total*_ the total numbers of events in the data set.

The average inter-rater agreement (Equation 3) across the 10 labeled EEG recordings was 93.01% (SD = 2.83%, min. = 87.65%, max. = 96.41%; absolute numbers can be found below). This indicates a high degree of agreement between raters and confirms that they can reliably distinguish between SOs and non-SO events.

A brief manual examination of cases where the two raters disagreed revealed two main reasons of disagreement. In most cases, one of the raters wrongly classified an event as being either an SO or a non-SO, presumably due to accidentally misclicking. In other cases, the event was ambiguous and the two raters had different internal thresholds for classification.

Nevertheless, as the average inter-rater agreement was very high, giving us a large sample of labels agreed upon by both raters, we only included these events in all subsequent analyses. In total, there were 96,277 events included (57,936 SOs, 38,341 non-SO).

### 2.3 Automatic SO detection algorithms

Three algorithms that have been previously used in the literature for automatically detecting SOs in human sleep data were included in this work and applied for detecting SO events in all EEG channels. A general overview of the criteria used by these algorithms can be found in Table 1. For simplicity, we have restricted the choice of the filter to a 2^nd^ order Butterworth filter. All algorithms were applied to the preprocessed EEG data in the same manner in which they have been previously applied in the literature (Massimini et al., 2004; Mölle et al., 2009; Ladenbauer et al., 2017).

**Table 1:** Summary of parameters and criteria used by the three automatic detection algorithms analyzed.

**Algorithm**	**Filter type**	**Freq. (Hz)**	**Dur.crit. (s)**	**Peak-to-peak amplitude (μV)**	**Neg. peak (μV)**
Absolute (Massimini et al., 2004)	2^nd^ order Butterworth bandpass	0.1–4	0.3–1	>70	< −40
Relative (Mölle et al., 2009)	2^*nd*^ order Butterworth bandpass	0.1–2	0.9–2	>2/3 of avg. amplitude	< 1/3 of avg. amplitude
Percentile (Ladenbauer et al., 2017)	2^nd^ order Butterworth bandpass	0.16–1.25	0.8–2	Largest 25%	–

The first algorithm under consideration (termed “absolute”) was introduced in Massimini et al. (2004). In brief, the preprocessed data are filtered between 0.1 and 4 Hz using a 2^nd^ order Butterworth bandpass filter and zero-crossings are identified. In the original algorithm, an event is considered an SO if a positive-to-negative zero-crossing and the following negative-to-positive zero-crossing are separated by 0.3–1 s, the negative peak between these two zero-crossings is smaller than −80 μV and the negative-to-positive peak-to-peak amplitude is larger than 140 μV. As EEG amplitude is known to significantly decrease with age (Leissner et al., 1970; Dijk et al., 2000; Segalowitz and Davies, 2004; Esser et al., 2007; Vyazovskiy et al., 2009; Dubé et al., 2015; Muehlroth and Werkle-Bergner, 2020), we adjusted these thresholds according to values previously used in the literature (Muehlroth and Werkle-Bergner, 2020), as follows: the negative peak threshold was decreased to −40μV, while the threshold for the peak-to-peak amplitude was decreased to 70μV.

For the second algorithm (termed “relative”), presented in Mölle et al. (2009), data were filtered again using a 2^nd^ order Butterworth bandpass filter with a frequency range of 0.1–2 Hz. Following zero-crossing identification, the average negative-to-positive peak-to-peak amplitude, and the average voltage value of the negative peak were calculated across all events defined by a separation of two consecutive positive-to-negative zero-crossings between 0.9 and 2 s. All events with an amplitude larger than two-thirds of the average amplitude and a negative peak value smaller than one-third of the average negative peak value were marked as SOs.

The third algorithm (Ladenbauer et al., 2017) (termed “percentile”) requires data to be bandpass-filtered between 0.16 and 1.25 Hz. All events defined by a separation of two consecutive positive-to-negative zero-crossings between 0.8 and 2 s are then selected and ordered according to the negative-to-positive peak-to-peak amplitude. The events with the 25% largest negative-to-positive peak-to-peak amplitude are marked as SOs.

All algorithms were implemented in *Sleepy* and are available in Donle et al. (2022).

### 2.4 Machine learning based detection algorithms

To test whether approaches that do not require parameters explicitly set by medical experts could perform better than previously described algorithms in detecting SO events, three different classes of machine learning based detection algorithms were investigated. First, the combination of a dynamic time warping (DTW) algorithm with a one-nearest-neighbor (1NN) classifier (Tan et al., 2017) (abbreviated by DTW-1NN in the following) was investigated, as this algorithm has been successfully used in speech recognition and other time series recognition tasks (Sakoe, 1971; Sakoe and Chiba, 1978; Keogh and Ratanamahatana, 2005; Bagnall and Lines, 2014; Bagnall et al., 2015; Silva and Batista, 2016). Second, combinations of feature generation, feature selection methods, and five classification machine learning models (logistic regression, decision tree, random forest, support vector machine, and multilayer perceptron with two layers) were investigated, all of which require the least computational and implementational effort. These methods together with DTW-1NN will be called machine learning methods in the following. Third, deep learning methods were investigated, since these algorithms typically show superior classification performances. For each method the same performance evaluation was conducted and the custom Python implementation of the best algorithm of each class was made available in our software tool *Sleepy*.

#### 2.4.1 Dynamic time warping with one-nearest neighbor classifier

First, we used a DTW-1NN algorithm. This approach has been successfully used in the field of speech recognition and other time series recognition tasks and is extensively described elsewhere (Sakoe, 1971; Sakoe and Chiba, 1978; Keogh and Ratanamahatana, 2005; Bagnall and Lines, 2014; Bagnall et al., 2015; Silva and Batista, 2016). In brief, DTW is a dynamic programming method used to find the optimal alignment between two time series (Keogh and Ratanamahatana, 2005). To that end, one of the series is warped in time and the optimal alignment between the two sequences is determined by minimizing the Euclidean distance between them. A full formal description of the method can be found in Keogh and Ratanamahatana (2005). The output, i.e., the DTW distance between time series, can subsequently be used as input for a 1NN classifier, a simple non-parametric learning algorithm which has been shown to be highly effective for classification (Bagnall and Lines, 2014; Bagnall et al., 2015; Silva and Batista, 2016). In this case, a query time series is classified by assigning to it the label of the training example nearest to it in terms of distance.

To avoid computing the DTW distance between a query time series and all time series in the training data set and thus reducing the computational complexity, we adopted a Time Series Indexing (Tan et al., 2017) (TSI) approach. To do so, a tree-like data structure is built from a small number of examples. The query examples are then classified using 1NN by comparing them to a set number of examples from the previously built tree structure.

During the classifier building step, TSI constructs a tree-like structure by recursively partitioning the data using *k*-means clustering. This is done by leveraging DTW Barycenter Averaging (Petitjean et al., 2011), a global averaging method for DTW, and subsequently associating time series to their closest centroids based on DTW. At every level of the tree, the data is split into a predefined number of clusters (10 in the current work) and the procedure continues recursively for the resulting clusters until the number of time series in each cluster is smaller than or equal to a predefined threshold (30 in the current work).

The second step involves classifying the query time series using the tree structure built in the first step and the 1NN search. The major advantage of TSI is that not all time series in the training data must be explored. The user can specify in advance how many time series should be seen in order to make a prediction for a query. As such, during nearest-neighbor search, the tree is explored from root to leaf, while maintaining three priority queues: one for the DTW distance and one for the lower bound Keogh (LB-Keogh; a computationally cheaper lower bounding measure which allows discarding of potential matches without the need for computing the full DTW), which store potential branches to explore once the current branch has been fully traversed, and a third one for the nearest neighbors. At every level of the tree, the distance between the query time series and the centroids at that level are calculated, and the algorithm continues exploring the branch closest to the query, while enqueueing the other ones in the two priority queues. Once a leaf has been reached, a similar logic is applied to all time series in that leaf: the DTW distance is computed between the query and all time series which could not be excluded based on the LB-Keogh. If the number of time series to be seen is reached, the algorithm terminates. Otherwise, it proceeds with the next branch in the queue. As it was unclear how the classification accuracy would depend on the number of time series seen in our case, we explored 13 values for this parameter, from 100 to 1,300 examples seen in steps of 100, all with a sampling rate of 500 Hz. A full description of the performance evaluation of this method can be found in Section 2.5.2.

Additionally, as it is common practice (Keogh and Ratanamahatana, 2005), we use a warping window (i.e., a locality constraint, which restricts the mapping of a time point in one time series to the specified window in the other time series) for constraining the DTW warping path, to prevent abnormal mappings, such as mapping most of one time series to a single point in the other time series, and to further increase computational efficiency. The length of the warping window was chosen to be 10% of the longest time series in our data set, as this has been proven to yield optimal results (Tan et al., 2018).

Further algorithmic details regarding the TSI implementation can be found in Tan et al. (2017). Our custom Python implementation in *Sleepy* (Donle et al., 2022).

#### 2.4.2 Machine learning

For each possible SO sequence, a set of 17 characteristic features (see Supplementary Table S3) was generated as input parameters, and combinations of four feature selection and five classification machine learning methods were trained using the corresponding labels as output parameters. Univariate selection, feature importance, recursive feature elimination (RFE), and principal component analysis (PCA) were investigated as feature selection methods, each in combination with a classification model consisting of logistic regression (LogReg), decision tree (DTree), random forest (RF), support vector machine (SVM), and multi-layer perceptron (MLP) with two layers (input and output layers). For each combination of feature selection and classification method, each reduction possibility between 16 and one feature(s) was investigated and each classification model was optimized separately for each feature selection method using a grid search. Thus, each combination of feature selection method, selected number of features (1–16), and hyperparameter-optimized classification model was examined individually. In addition, an approach without feature selection was also examined. This resulted in 325 evaluated approaches from which the best approach was selected. Additionally, as suggested in the literature (Kate, 2015), a DTW feature construction method was investigated in combination with each hyperparameter-optimized classification model. This method uses the DTW distance to a few selected sequences as features for the classification. In the current study, hierarchical clustering was used to identify ~500 characteristic sequences from the data set, which were then used to calculate the distance to a new unidentified sequence. These DTW distances were then used as features for the following classification, which was performed in the same manner as in the previous section. All Machine Learning approaches were implemented in Python using Scikit-learn.

#### 2.4.3 Deep learning

End-to-end deep learning models (DeepL) allow to skip the feature generation step and use the EEG sequence directly as input. The investigated general architectures include:

- Convolutional Neural Networks (CNN),
- Long Short-Term Memory Networks (LSTM),
- Convolutional Layers as input to an LSTM Network (CNN + LSTM),
- Bidirectional Long Short-Term Memory Networks (BiLSTM),
- A network consisting of a layer that combines convolutional aspects with LSTM networks (ConvLSTM) (Shi et al., 2015).

**Figure 2:**
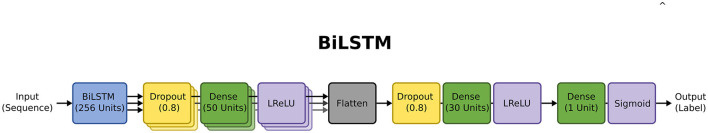
Model structure of the best performing architecture (BiLSTM) with optimized hyperparameters.

For some hyperparameters (see Supplementary Table S4) of the Deep Learning networks, generally recommended values were taken from the literature (Kingma and Ba, 2015; Smith, 2015; Janocha and Czarnecki, 2017; Smith et al., 2017; Kandel and Castelli, 2020). For the remaining hyperparameters (see Supplementary Figure S3), a random search was performed with 100 instances to find a good initialization. Then, structural hyperparameters of the networks were optimized from front to back, i.e., the architecture was optimized layer by layer starting with the first layer. Finally, general hyperparameters, such as the choice of optimizer, were explored. For each hyperparameter set, a customized five-fold cross-validation was performed, where only three of the five combinations were randomly selected to reduce computational effort while compensating for random events in the training phase. After the hyperparameter search, which was performed separately for each of the five general architectures, all five approaches were compared and the best architecture (BiLSTM) was selected. The model structure of this network is shown in Figure 2. The other optimized architectures and the exact hyperparameter values are shown in the Section 1.4 in Supplementary material. All Deep Learning models were implemented in Python using Keras and GPU utilization with NVIDIA CUDA (NVIDIA et al., 2020).

### 2.5 Performance evaluation

#### 2.5.1 Performance of the automatic SO detection algorithms

In order to compare the performance of the automatic SO detection algorithms described in Section 2.3 against the manually labeled data, we calculated the balanced accuracy (Equation 4) of each of the three automatic SO detection algorithms on the manually labeled data set. Balanced accuracy (BAcc) was defined as:


(4) $$\begin{array}{l} BAcc=0.5\cdot \left(TPR+FPR\right)=0.5\cdot \left(\frac{TP}{TP+FN}+\frac{TN}{TN+FP}\right), \end{array}$$

where TP is the number of true positives, TN is the number of true negatives, FP is the number of false positives, and FN is the number of false negatives.

Due to different bandpass filter values (see Table 1 and Section 2.2.2) used across the three automatic detection algorithms and in the manually labeled data set, the event start and end points were not identical across the algorithms. Therefore, to determine the performance of each automatic detection algorithm on the manually labeled data set, for all events detected by an automatic detection algorithm, we considered an event to be a true positive if there was at least 50% overlap with a manually identified SO event and a false positive otherwise. Accordingly, a non-SO event from the manually labeled data set was considered a true negative if there was no corresponding event detected by the automatic detection algorithms with at least 50% overlap. An SO event not detected by the automatic detection algorithms was counted as a false negative.

#### 2.5.2 Performance of machine learning based detection algorithms

The data, consisting of the prefiltered sequences and the manual labels of those sequences for which both raters agreed on the same label, were divided into training, testing, and validation data for all three classes of methods. To prevent overfitting, data from five participants were split into training and testing data, and the data from the remaining five participants (54,515 examples) were kept for a first validation data set (V1; Table 2; an overview of all steps conducted for obtaining data set V1 and the other two data sets referenced below can be found in Supplementary Figure S2). The training data used for building and training the classification methods had a size of 5% (2,088 examples) for the DTW-1NN method and 70% (29,233 examples) for all machine learning and deep learning methods. Respectively, the test data had a size of 95% (39,674 examples) and 30% (12,529 examples). For comparison of the methods the training and testing procedure was conducted ten times, one for each of the shuffled training sets as part of the 10-fold cross-validation and the performances on the test set were averaged.

**Table 2:** Overview of the three validation data sets.

**Data set**	**Prefiltering**	**Manual inspection**	**# examples**
V1	Yes	Yes	54,515
V2	No	No	200,350
V3	No	Yes	13,903

The prefiltering step described in detail in Section 2.2.1 was only applied prior to the manual inspection of data set V1. Data set V2 includes all events with durations between 0.8 and 3.5 s for the five validation participants. We use the same event labels obtained through manual inspection for V1, marking as non-SOs the events not seen by medical experts in data set V1.

In addition, two further validation data sets were used to evaluate the performance of the methods independent of pre-filtering. Of the five validation participants, all events with durations between 0.8 and 3.5s (200,350 examples) were selected as the second validation data set (V2) and sequences from a single channel of all ten participants that were labeled by medical experts before the pre-filtering procedure (13,903 examples) while excluding the training sequence of these channels were selected as the third validation data set (V3). Note, however, that V2 contains some true positive events that were considered as “non-SO” by the pre-filtering procedure and were thus not seen by medical experts (see Section 2.2.1 and Supplementary Table S2).

## 3 Results

### 3.1 Semi-automatic SO labeling tool

We developed a platform-independent Python semi-automatic SO labeling tool and a pre-filtering procedure in order to be able to label SO events in EEG data in a standardized and time-efficient manner. As SO events are relatively sparse, both the tool and the pre-filtering procedure use this property to select and show the user only those events which are more likely to be SOs. This considerably speeds up the labeling time without pre-filtering. Our raters needed ~8 h to inspect a channel from an ~90-min time series, which would translate into ~224 h for one recording with 28 channels. When *Sleepy* presented only the pre-filtered data, raters needed only ~30 min for a single channel recording of 90 minu. This translates into a 16-fold speed increase for manually labeling SOs in sleep EEG. This speed improvement comes with the trade-off that some SO events (up to 20% for some participants) are lost due to the pre-filtering. Nevertheless, in our case, up to 97% of the non-SO events are also successfully removed (see Supplementary Table S2).

As input data, the tool accepts  .mat EEG files preprocessed with the software package FieldTrip in its standard FieldTrip data structure. Output data is written in the same file format. Pre-filtering parameters can be set in a graphical user interface [available in *Sleepy* (Donle et al., 2022)].

**Figure 3:**
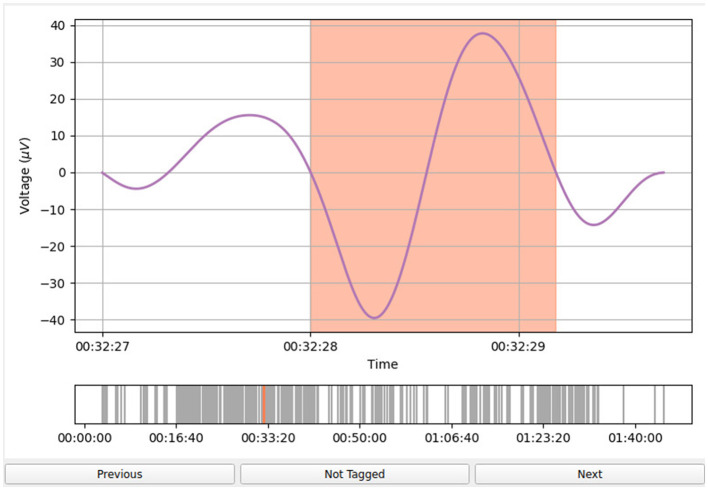
*Sleepy* graphical user interface. The event to be inspected is highlighted in orange in the EEG time series. On the *x*-axis, the rater can see the time stamp of the event relative to the beginning of the EEG recording, while on the y-axis, the voltage [μV] is shown. The “Not Tagged” button indicates that the event has not been tagged as a false positive. If the user presses this button, the label becomes “Tagged” and the button appear colored in red to indicate the user's choice. Inspecting the next event or inspecting the previous event is possible by pressing “Next” or “Previous” buttons, respectively.

An example of the user interface of *Sleepy* is shown in Figure 3. The event being inspected (in this case, an SO event obtained after applying the pre-filtering procedure outlined in Section 2.2.1) is highlighted in orange and several seconds of the EEG time series are displayed both before and after the event to give the raters more context for the selected event. A second panel shows all detected events of a particular channel marked as gray lines on the time axis.

By default, all events are considered true positives. For every event, the user has the option of marking it as a false positive by pressing the “Not Tagged” button, inspecting the next event, or inspecting the previous event.

### 3.2 Automatic detection algorithms

The total number of SO events detected by each of the three automatic SO detection algorithms is shown in Table 3. The *absolute* algorithm detects the lowest number of events, while the relative one has the highest count. This is in line with previous reports (Muehlroth and Werkle-Bergner, 2020), which indicate that algorithms using absolute amplitude thresholds tend to underestimate SO numbers in older adults compared to algorithms with individually adjusted thresholds. The difference between the relative and percentile algorithms can also be explained by the fact that the latter selects only the 25% largest amplitude SOs, while the former includes all events passing the individually adjusted threshold.

**Table 3:** Number of SO events identified by each of the three automatic detection algorithms across all participants and sleep stages and balanced accuracy of the three algorithms (relative, percentile and absolute) evaluated on the manually labeled data set obtained according to the procedure described in Sections 2.2.1 and 2.2.2.

**Algorithm**	**# SOs**	**Balanced accuracy**
Relative	157,571	56.87%
Percentile	89,521	61.03%
Absolute	41,608	58.48%

We evaluated the performance of the three automatic SO detection algorithms against the manually labeled data obtained according to the procedures described in Sections 2.2.1 and 2.2.2. For that, we calculated the balanced accuracy. It is important to reiterate here that we only considered those events for which there was a label in our manually created data set. A more detailed description of the performance evaluation can be found in Section 2.7.2. The results are shown in Table 3. All three algorithms have a relatively low performance on the manually labeled data set (maximum balanced accuracy: 61.08%). Although the differences between them are small, the percentile algorithm achieves the best performance. The relative algorithm gives the poorest results due to the high number of false positives.

To account for a possible bias induced by the fact that the automatic detection algorithms were developed based on different SO definitions, we conducted a comprehensive grid search over the free parameters of these algorithms, which revealed that balanced accuracy only slightly improves under certain parameter combinations. More specifically, the best accuracy for the percentile algorithm was 58.99% (down from 61.03%), for the relative algorithm it was 57.10% (up from 56.87%), and for the absolute algorithm it was 64.42% (up from 58.48%). For the first two algorithms, we optimized over the lower and upper filter boundaries (0.1–0.9 Hz in steps of 0.2 Hz, 1–4 in steps of 0.5 Hz) and over the lower and upper SO duration boundaries (0.2–0.8 s in steps of 0.2 s, 1–2 s in steps of 0.2 s). For the absolute algorithm, we optimized over the same filter values, over the separation of zero-crossings (0.2–1.4 s in steps of 0.2s), and over the peak-to-peak and negative peak amplitude (20–80 μV in steps of 20 μV and 20–60 μV in steps of 20 μV).

Although the three algorithms show a quantitatively low performance, we wanted to check whether the qualitative results obtained through either the automatic detection algorithms or the manual labeling procedure differ. The reason is that one could still draw meaningful conclusions regarding, for example, relative differences between sleep stages (e.g., higher SO amplitude and density in deeper sleep stages compared to lighter ones), in spite of the apparent quantitative differences. For that, we chose two commonly used SO measures: density and amplitude. These measures were applied to all events detected by the automatic SO detection algorithms in the preprocessed EEG recordings, as well as to the manually labeled data set. In terms of density, all three automatic detection algorithms, as well as the manual labeling, detected the highest number of events in sleep stage N3, followed by stages N2, N1, and awake (Supplementary Figure S3). This indicates a preservation of the common pattern of increasing numbers of SOs with increasing sleep depth regardless of the detection method. In terms of density over- and underestimation within the same sleep stage, one needs to take into account the total number of events detected by each of the three algorithms (shown in Table 3) in comparison to the total number of manually labeled SO events (57,936 events). The same pattern (relative, followed by percentile, manual, and absolute) observed in the total number of events is reflected in density as well.

The average amplitude and overall morphology of detected SO events (Supplementary Figure S4) was similar for the manually labeled data, as well as the relative and percentile algorithms in all three sleep stages. In the awake period, the average amplitude in the manually labeled data was approximately twice that of the relative and percentile algorithms. The absolute algorithm showed the largest amplitude in all sleep stages and the awake period. The absolute algorithm imposes a conservative amplitude threshold, thus selecting only the highest amplitude events, while both the relative and percentile algorithms are adjusted to each individual, which explains the current results.

### 3.3 Machine learning based detection algorithms

#### 3.3.1 DTW-1NN

To improve classification of SO and non-SO events in sleep EEG data, we used a DTW-1NN classifer. To speed up classification of the events, the user can specify the number of examples to be used for classification. As this number was not known *a priori*, we tested a range of possible values, from 100 to 1,300 in steps of 100. **Figure 6** shows the balanced accuracy of the 10-fold cross-validation procedure (conducted by randomly splitting the data into 5% for building the classifier and 95% for evaluating its performance; see Section 2.5.2 for details) as a function of the number of examples seen from the training data set (see Section 2.5.2). Increasing the number of examples seen increases the balanced accuracy and decreases the variance across folds. Beyond 1,000 examples seen, the balanced accuracy increases and the variance decreases only minimally (95.10% ± 0.460 for 1,000 examples seen vs. 95.26% ± 0.360 for 1,100 examples), which is why this value was selected for classifying the events from the validation data set.

Table 4 summarizes the results in terms of balanced accuracy of the DTW-1NN with 1,000 training examples seen for our three validation data sets obtained from the manually labeled data and described in detail in Section 2.5.2. On the first validation data set V1 (i.e., pre-filtered data which was not included in the training data set), the balanced accuracy remained comparable to the testing values (95.98% for V1 vs. 95.10% for the testing data set). For the second data set, V2, where the pre-filtering step was not applied (see Section 2.5.2.), the balanced accuracy dropped to 87.69%. This was expected, as this data set includes, on the one hand, true SOs which were excluded in the pre-filtering step, and on the other hand, more false positives. Finally, on the third data set, containing only those channels fully labeled by manual experts, the balanced accuracy was 82.15%.

**Table 4:** Balanced accuracy of the DTW-1NN, RF + RFE, and BiLSTM algorithms across different validation data sets.

**Data set**	**DTW-1NN**	**RF + RFE**	**BiLSTM**
V1	95.98%	98.61%	99.20%
V2	87.69%	89.19%	89.81%
V3	82.15%	83.142%	87.89%

The classifiers were built on the data described in Section 2.5.2. A full description of the three data sets (V1, V2, V3) can be found in Section 2.5.2.

**Figure 4:**
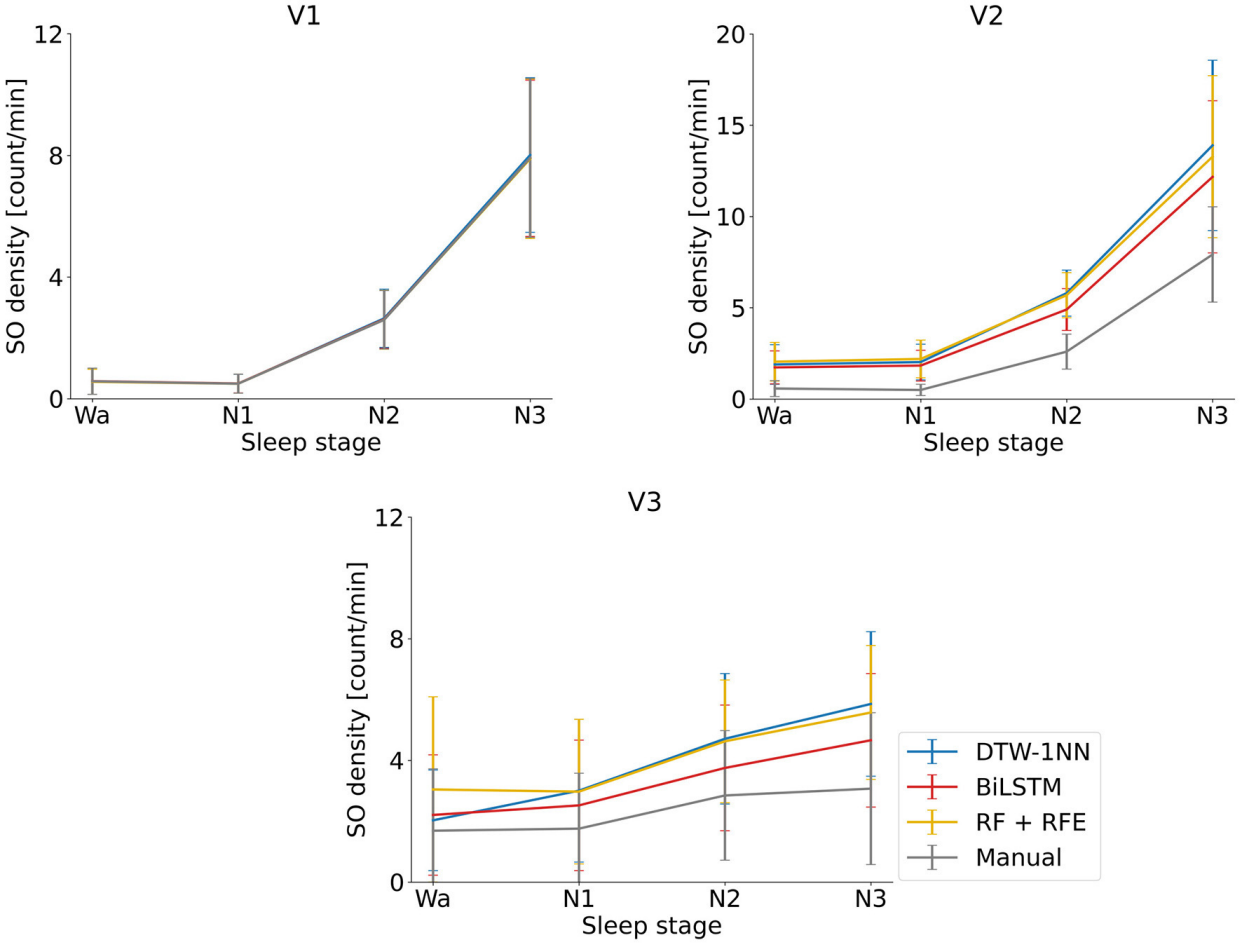
Average and standard deviation of the SO density (number of SOs per minute) across participants for each the three ML/DL methods developed in this work (DTW-1NN, RF + RFE, BiLSTM). The algorithms were applied to the three data sets (V1, V2, V3) described in Section 2.5.2. and the manually labeled data set obtained according to the procedure described in Sections 2.2.1. and 2.2.2. Results are shown for each of the three sleep stages (N1–N3) and for the awake state (Wa).

**Figure 5:**
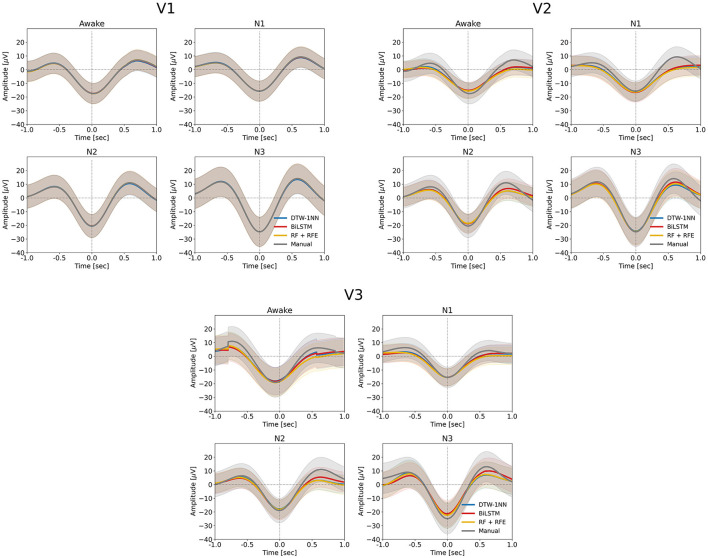
Average SO waveform for each the three ML/DL methods developed in this work (DTW-1NN, RF + RFE, BiLSTM) and for the manually labeled data set obtained according to the procedure described in Sections 2.2.1. and 2.2.2. for each of the three sleep stages (N1–N3) and the awake state (Wa). The solid lines mark the average negative-to-positive peak-to-peak amplitude for each of the three ML/DL methods (blue—DTW-1NN, red—BiLSTM, yellow—RF + RFE) and the manually labeled data (gray), while the shaded area represents the standard deviation around the mean. In each of the four cases (awake state and sleep stages N1–N3), all three algorithms closely track the manually labeled data. The amplitude of the detected events is largest in sleep stage N3 compared to the other sleep stages for all three algorithms and for the manually labeled data.

We looked at descriptive SO statistics (density and amplitude) across the SO events identified by the DTW-1NN algorithm for all three data sets (V1, V2, and V3). As shown in Figure 4, there is no difference in SO density between the DTW-1NN algorithm and the manually labeled data in V1, whereas in V2 and V3, DTW-1NN overestimates the number of SOs present in the data. The SO amplitude is similar between DTW-1NN and the manually labeled data across all sleep stages in all data sets (Figure 5).

#### 3.3.2 Machine learning

In Table 5, the averaged results in balanced accuracy over the test data sets of the 10-fold cross-validation are shown for different combinations of feature selection and classification method. For each combination, 16 results are available, one for each number of selected features, but only the best result is shown, and the corresponding number of selected features is given in brackets. It is obvious from the results that the different feature selection methods only lead to a small improvement in accuracy compared to the approach without feature selection, indicating that most of the features hold significance and selection is not advantageous. This is also indicated by the fact that in many cases the selection of 16 features gave the best prediction results. The random forest classification method provides the highest prediction accuracy in most cases and the decision tree works well also with a small number of features. The DTW feature construction approach performs worse than the classical feature engineering approach. However, the combination of feature importance or recursive feature elimination (RFE) as a feature selection method and the random forest as a classification model provides the highest prediction accuracy.

**Table 5:** Results on the averaged results on the test set for each combination of feature selection and classification method in balanced accuracy.

**Method**	**LogReg**	**MLP**	**DTree**	**SVM**	**RF**
No feature selection	96.75% (17)	97.89% (17)	98.66% (17)	97.96% (17)	98.78% (17)
Univariate selection	96.75% (16)	98.23% (9)	98.66% (7)	98.11% (11)	98.83% (16)
Feature importance	96.75% (15)	98.29% (8)	98.66% (7)	98.10% (7)	**98.83%** (16)
RFE	96.75% (10)	98.43% (13)	98.66% (4)	97.54% (15)	**98.83%** (16)
PCA	96.88% (11)	98.46% (15)	93.82% (10)	97.60% (12)	98.29% (16)
DTW feature const.	95.14%	93.98%	94.20%	93.20%	94.72%

Values in bold indicate the highest accuracy reached overall. The corresponding number of selected features out of the 17 in total is indicated in parentheses. Additionally, the results of the DTW feature construction (without feature selection) with each classification method in balanced accuracy is shown.

The prediction results in terms of balanced accuracy of the random forest classification combined with RFE as the best performing ML approach for the three validation data sets can be seen in Table 4. For the first validation data set V1, the prediction results of 98.61% are comparable to the test data sets results of 98.83% and show that the results from the test phase are transferable to unknown participants. Prediction results decreased to 89.19% for the second validation data set V2 (i.e., all participants, no pre-filtering) and to 83.142% for the third validation data set V3. The pattern of performance on all three validation data sets is comparable to the performance of the DTW-1NN approach, only with slightly higher accuracies and lower variance.

The descriptive SO statistics (Figures 4, 5) are similar to those described for the DTW-1NN case.

#### 3.3.3 Deep learning

The performance of the different optimized Deep Learning architectures were evaluated on the five test data sets of the five-fold cross validation, with the results shown in Table 6. BiLSTM was the architecture which produced the best results, closely followed by the LSTM architecture. Therefore, the BiLSTM model was chosen as the best DeepL approach.

**Table 6:** Results on the five test data sets of the five-fold cross-validation in balanced accuracy for different deep learning architectures individually optimized for hyperparameters.

**Architecture**	**Balanced accuracy ±SD**
CNN	98.58% ± 0.217%
LSTM	99.20% ± 0.087%
CNN + LSTM	98.28% ± 0.504%
BiLSTM	99.32% ± 0.051%
ConvLSTM	98.64% ± 0.707%

Table 4 shows the balanced accuracy of the best performing model on the three validation data sets. The relative trend of the results of the BiLSTM model was once again very similar to the DTW-1NN and ML approaches, but with overall higher values. The results for the first validation data set V1 (see Section 2.5.2.) gave a balanced accuracy of 99.20%, which is also very similar to the results for the test data set of 99.32% and once again indicates that the results of these methods are well transferable to unknown participant of the same age group and with the same experimental setup. This assumption can also be inferred from the small standard deviations of the results across the different runs of the 10-fold cross-validation. For the validation data sets V2 and V3, the prediction results dropped to 89.81 and 87.89%, respectively. Thus, the BiLSTM approach has the highest predictive performance in all training and validation data sets.

The descriptive SO statistics (Figures 4, 5) are similar to those described above for the DTW-1NN and RF + RFE cases. Nevertheless, in particular regarding SO density, the BiLSTM algorithm is closest to the manually labeled data set.

### 3.4 Computation time

Table 7 shows the computation time for training (if any) and prediction for different methods on the same computing platform. Since platform specifications have a major impact on computation time, these results should only be considered in relation to each other or as rough estimates. The training time is the time taken to train a single model using the amount of training data specified in Section 2.5 or the amount mentioned in the brackets, and the prediction time is the time taken to predict the labels of 6,000 putative SO sequences, which is approximately the number of sequences recorded during 1 h of EEG measurement with 28 channels.

**Table 7:** Comparison of computation time for training (if any) and prediction for different methods, where training time is the time taken to train a single model using the training data of ~40,000 sequences, and prediction time is the time taken to predict the labels of 6,000 sequences on a Intel i7-11600 CPU and NVIDIA GeForce GTX 1660 GPU.

**Method**	**Training time**	**Prediction time**
Automatic SO detection	None	~1 min
DTW-1NN (100 seq. seen)	2 h 50 min	51 min 48 s
DTW-1NN (1,000 seq. seen)	2 h 50 min	2 h 5 min
ML (RF + RFE)	1 min 20s	< 1s
DeepL (BiLSTM)	26 min 9s	< 1s

The automatic SO detection algorithms require no training and the prediction time is short (~1 min for 6,000 sequences). The RF + RFE and the BiLSTM methods have the shortest prediction time, with < 1 s required for classifying the same amount of events. The training time is longer for the BiLSTM method (26 min 9 s) compared to the RF + RFE method (1 min 20 s), but it remains relatively short. In constrast, the DTW-1NN method requires both the longest training (2 h 50 min) and the longest prediction time. As the classifier is built only once (see Section 2.4.1), the training time shown in the table is identical for both numbers of sequences seen. Building the DTW-1NN classifier requires a long time, as computing the DTW distance is time-intensive. During the prediction step, the time necessary for classifying 6,000 events using DTW-1NN increases as the number of training sequences to be seen is higher (51 min 48 s for 100 sequences seen, 2 h 5 min for 1,000 sequences seen). These results, combined with the performance of the algorithms (Table 4) suggest that the BiLSTM method is the best method of choice, as it combines the lowest prediction time with the highest accuracy, while keeping training time modest. Nevertheless, the BiLSTM method also requires the usage of a GPU, which might not be readily available on common setups. In this case, the RF + RFE method becomes optimal, as it still maintains a high accuracy (see Table 4), while requiring only a short training time on a CPU.

### 3.5 Effect of the number of training data on classifier performance

In Figure 7, the balanced accuracy regarding the test data sets of the three machine learning based approaches (DTW-1NN, Machine Learning and Deep Learning) are shown for different numbers of training samples. The machine learning approach (RF + RFE) provides the highest accuracy up to 3,000 training sequences, but beyond 3,000 training sequences, the BiLSTM deep learning model achieves the highest predictive power. Importantly, labeling only 3,000 sequences would require roughly 1.5 h of manual work.

**Figure 6:**
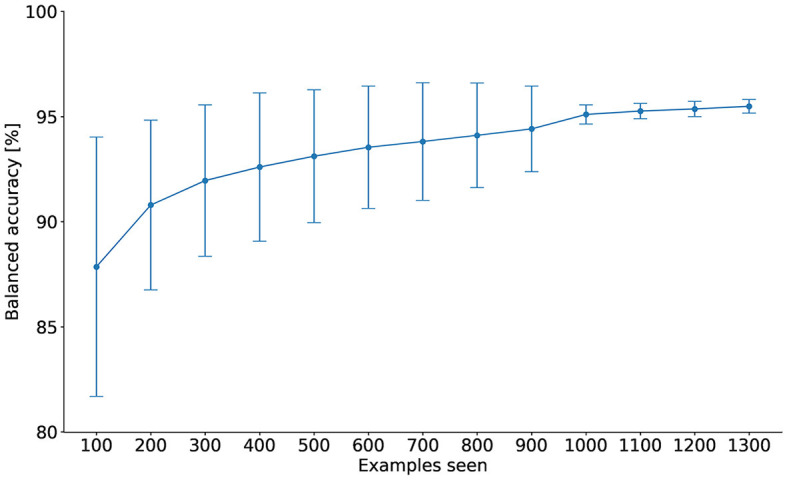
Average balanced accuracy and standard deviation for DTW-1NN as a function of the number of training examples seen. Starting from 1,000 examples, the variance across the 10 seeds significantly decreases, indicating that the algorithm is more stable, and the balanced accuracy increases only minimally.

**Figure 7:**
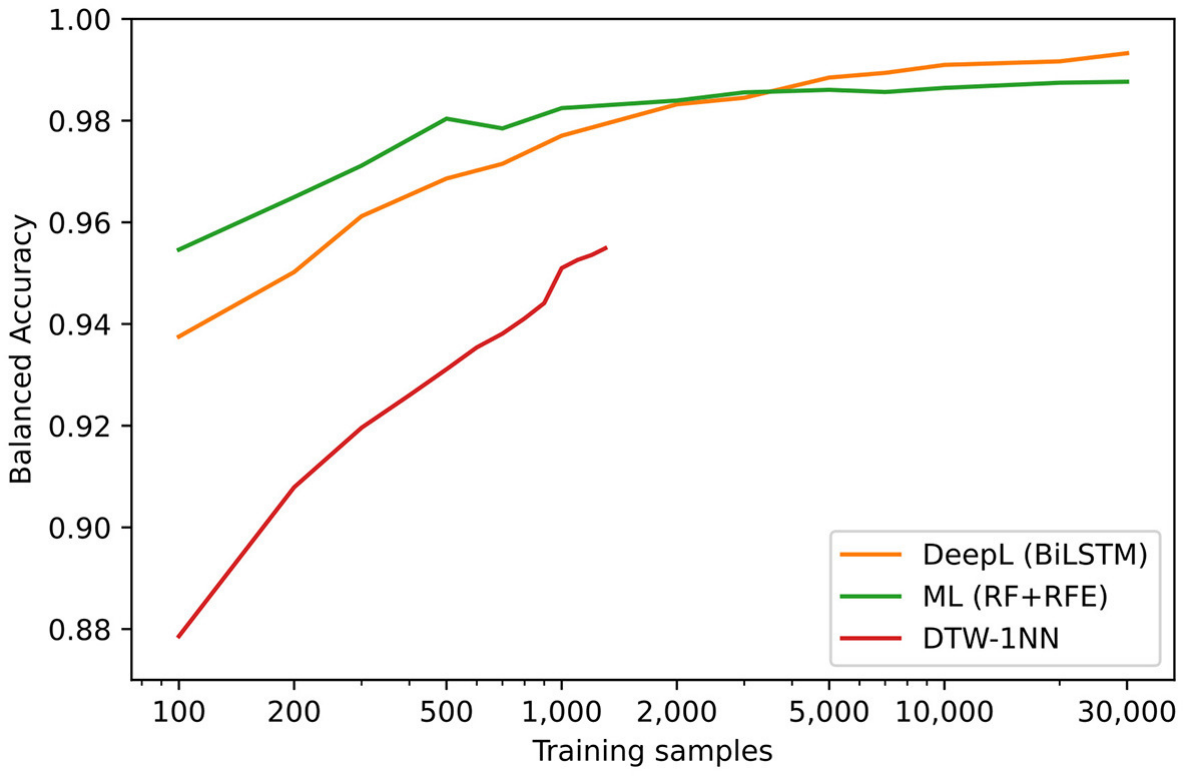
Results on the test data sets in terms of balanced accuracy for different methods and for different quantities of training samples. Since the prediction time of the DTW-1NN approach (red line) increases dramatically for a higher number of training samples, the number of training samples had to be limited to 1,300. For the deep learning approach (orange line) the BiLSTM architecture was used and for the Machine Learning approach (green line) the Random Forest Classifier with RFE was chosen.

### 3.6 Recommended workflow

**Figure 8:**
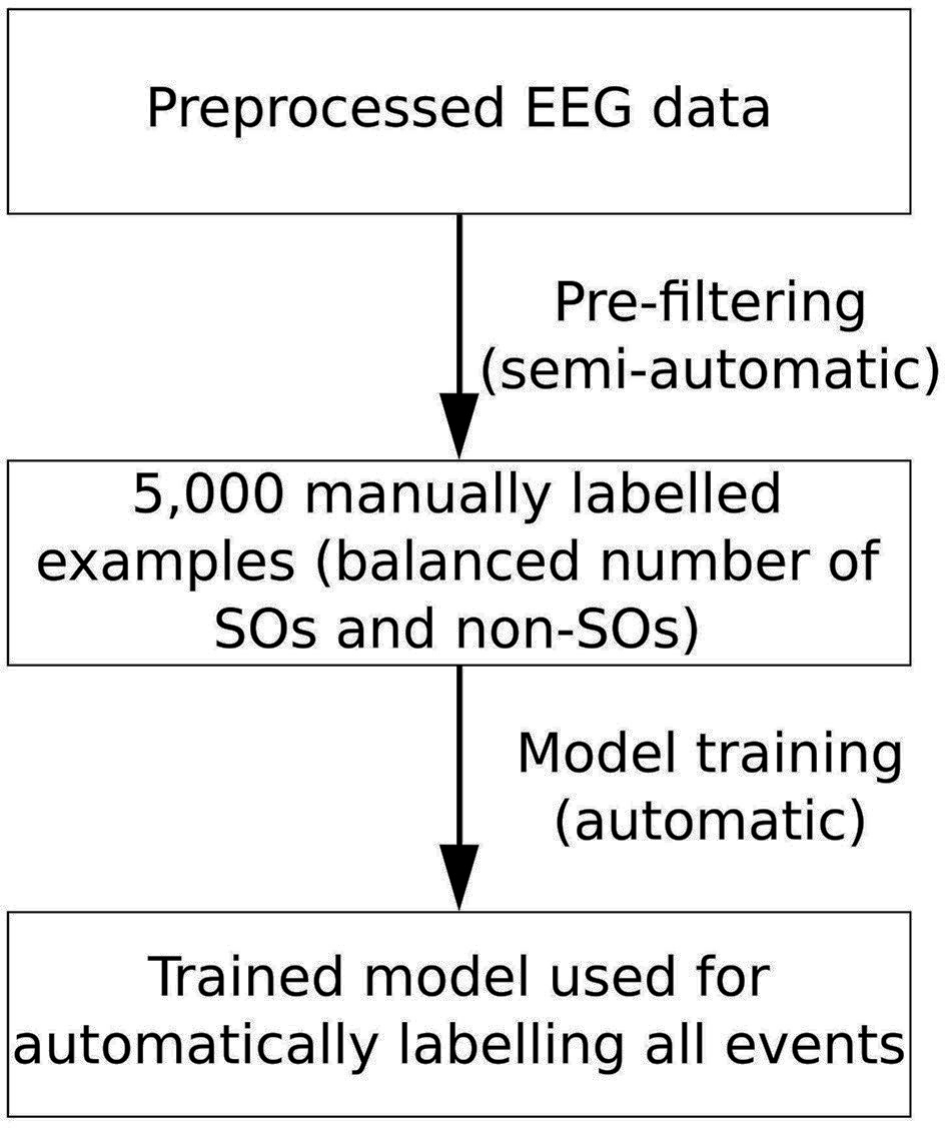
Workflow overview for using *sleepy* on a new EEG data set. We recommend first applying the pre-filtering procedure outlined in Section 2.2.1 to obtain a corpus of 5,000 manually labeled examples, aiming for a balanced number of SO and non-SO events. This step is the only one that requires manual input from the user and we estimate that it takes ~2.5 h. Subsequently, we recommend retraining the BiLSTM model (if a GPU is available) or the RF + RFE model (if a GPU is not available) on this set of events. The model thus trained can be then used to label all events in the data set.

As it is apparent from the results presented in the previous sections, all three machine learning based approaches proposed in this work generalize to the other participants in our sample (e.g., results on V1). Nevertheless, in the current work, we have only examined data from older adults which was acquired under the same experimental conditions. Additionally, the training data sets (i.e., manually labeled data after the pre-filtering procedure) are relatively balanced, with ~60% SO and ~40% non-SO events. In contrast, prior to pre-filtering, the number of non-SO events is significantly higher than that of SOs. This means that only labeling a small number of events, without applying the pre-filtering procedure, could result in only a very small number of SOs in the training data. Therefore, for optimal performance on a new group of participants, we recommend to first use our SO labeling tool for pre-filtering and manually labeling a set of examples, then train the BiLSTM algorithm (as this gives the highest performance) on this new set of examples and then use the trained model for labeling the entire data set. Importantly, should a GPU not be available for training the BiLSTM algorithm, the RF + RFE approach could be easily used instead and still achieve a significantly good performance (Figure 8).

Based on Figure 7, we recommend labeling of 5,000 examples (estimated work time: ~2.5 h) in order to achieve a close to optimal performance of the BiLSTM algorithm. The results for unknown participants (results for V1) suggest that the hyperparameter-tuned version of the BiLSTM, as shown in Figure 2 and explained in the Section 1.4 in Supplementary material, will be able to reproduce similar results for different age groups and experimental conditions when trained on this new data.

However, as labeling many examples can be time-consuming and accuracy remains relatively high even with fewer examples, one can use the information provided in Table 7 and Figure 7 to determine what would be an acceptable speed-accuracy trade-off and thus select a different algorithm with a lower required number of labeled training examples, e.g., 250 examples and the RF + RFE approach with a balanced accuracy of ~98%.

In summary, we recommend using the provided tool to generate a number of manually labeled examples from the data set of interest. In terms of optimal performance (both speed and accuracy), we recommend a BiLSTM approach with a minimum of 5,000 training examples. At the same time, we recognize that individual research requirements might differ, so we encourage using the information provided in Table 7 and Figure 7 for making an individually-tailored decision.

## 4 Discussion

In this work, we developed a semi-automatic tool for labeling SO events in sleep EEG data and used it to build the first validation data set of manually labeled SO and non-SO events. Additionally, we compared the performance of traditionally used automatic SO detection algorithms on our ground truth data set. Finally, we applied a DTW-1NN algorithm, as well as machine learning and deep learning techniques to the manually labeled validation data to improve SO detection.

Manual inspection of SO events in sleep EEG data is extremely time-consuming and not feasible for large data sets, and to the authors' best knowledge, data sets for benchmarking new SO detection algorithms are currently lacking. To considerably speed up this process and be able to create such a data set, we took advantage of the fact that SO events are relatively sparse and developed a pre-filtering procedure which only selects good SO candidates for manual inspection (see Sections 2.4, 3.1, and Supplementary Table S2). While up to 20% of SO events are lost during the pre-filtering procedure, up to 97% of false positives are also removed. This significantly speeds up the manual labeling procedure (up to 16 times in our case). To show that the loss of true positives does not significantly impact the generalization of machine learning and deep learning algorithms, the performance of these algorithms is tested on the channels manually labeled before the pre-filtering procedure, from which training examples are excluded (see Section 2.7). The results are discussed below.

We have shown that automatic SO detection algorithms based on both absolute and relative amplitude criteria give results qualitatively similar to those obtained from manual labeling in terms of SO density. SO amplitudes are similar between our manually labeled data and the events detected by adjustable threshold approaches, while absolute thresholds lead to higher amplitudes. As our sample of participants consisted of older adults, this confirms previous reports showing that absolute thresholds are too strict for these participants (Muehlroth and Werkle-Bergner, 2020). Most notably, however, quantitative results show that automatic SO detection algorithms perform relatively poorly on our ground truth data set, with the highest balanced accuracy reaching only 61.03%. It is important to note that previous automatic detection algorithms were developed based on different SO definitions and other experimental setups compared to the one used in the current work and that our novel methods were specifically tailored to our definition. Therefore, the current quantitative comparison has limited generalizability and the automatic detection algorithms could still perform better on other benchmarking data sets tailored to other SO definitions, such as overnight recordings from younger adults. Nevertheless, the optimization of the free parameters of these automatic algorithms did not lead to any significant increase in accuracy on the current data set. Hence our results underscore the need for improved SO detection algorithms for non-normative populations, which we have developed in this work.

The first approach, DTW-1NN, showed a very high accuracy both on the training and on the V1 data set (see Figure 6 and Table 4). The accuracy on the V2 and V3 data sets dropped, but remained much higher compared to that of the automatic SO detection algorithms. The accuracy on these two data sets remained above 80%, indicating a good classification performance. Nevertheless, the drop in accuracy is to be expected, on the one hand because the classifier was not built on these two data sets, and on the other hand because of the true positive events discarded by the pre-filtering procedure. Although this represents an improvement compared to traditional SO detection methods, the drop in accuracy coupled with the relatively long time necessary for classification prompted us to explore additional classification techniques.

The machine learning approaches had higher accuracy on test and validation data sets than DTW-1NN, with a very similar relative trend of prediction results, while drastically reducing computation time. More specifically, the RF + RFE machine learning approach achieved an accuracy of 98.83%, while requiring only minimal CPU training time.

While deep learning models, especially the BiLSTM architectures, provided the best prediction results, the implementation effort, especially hyperparameter search, was significantly higher. However, it is reasonable to assume that it is possible to re-train the model with the same hyperparameters on a different data set to save some implementation effort and get similarly good results.

It has also been shown that with more data better results are achieved, but that even with small amounts of data the obtained results are comparatively good. For example, machine learning models can still perform well using < 3,000 training sequences, achieving a balanced accuracy of more than 98%. In general, it is apparent from the results that deep learning models show better performance with increasing size of the training data set and that multi-participant models perform better than single-participant models (Section S1.6 in the Supplementary material).

In terms of SO density, all three approaches (DTW-1NN, RF + RFE, and BiLSTM) give results almost identical to the manually labeled data on data set V1 (Figure 4). This can be explained by the fact that the accuracy in this case is larger than 95% for all three algorithms. As the accuracy decreases on data sets V2 and V3, it becomes apparent that the three algorithms developed in this work tend to overestimate the number of SOs detected. The BiLSTM algorithm, which has the highest accuracy on these data sets, performs closest to the manually labeled data. Regarding SO amplitude, there is no significant difference between our developed algorithms and the manually labeled data on any of the three data sets, suggesting that the events detected by the algorithms are similar in shape to the other SO events (Figure 5).

One limitation of the current study is the fact that the data were referenced to an electrode attached to the nose instead of using the standard mastoid reference. This choice was imposed by the experimental setup, in which the mastoid electrodes were used to deliver transcranial current stimulation and could thus not be used for referencing purposes. As two of the automatic detection algorithms (Massimini et al., 2004; Mölle et al., 2009) examined in the current work were developed using EEG signals referenced to the mastoids and as the choice of reference impacts SO characteristics (Mensen et al., 2016), the possible impact of EEG reference on SO detection cannot be clearly assessed here. Another limitation is that we used only one data set, recorded from older adults during an afternoon nap. As previous studies suggest, SO events show differences between older and younger adults (Muehlroth and Werkle-Bergner, 2020). Additionally, some studies record EEG data during nighttime sleep instead of during the afternoon. Therefore, while the procedure outlined in this work can be used to manually label training examples in a new data set and re-train the algorithms we have developed, it would be interesting to see if the algorithms trained on the current data set could generalize to others, as this would speed up the SO detection procedure. Given that SOs recorded during nighttime sleep of younger adults are more easily differentiable, showing higher amplitude and a more stereotypical shape (Muehlroth and Werkle-Bergner, 2020), we expect that to be the case. Finally, while electrodes F3 and F4 are typically the strongest SO detection electrode sites, in the current work, they were excluded for two out of ten participants due to the fact that they were used to deliver transcranial stimulation. Including these electrodes in the analysis could potentially improve the detection accuracy.

While the current study lays the groundwork for benchmarking SO detection algorithms, we plan to extend our tool by including support not only for * .mat* files, but for all commonly used EEG data formats. Additionally, we are planning on validating our current algorithms on different data sets acquired under different conditions, as mentioned above. The tool can also be augmented to display the percentage of SOs in a given epoch, thus having the potential of being used for facilitating sleep staging by sleep technologists.

In conclusion, in this work we have developed a tool for manually labeling SO events in sleep EEG data in a standardized and time-effective manner. Using this tool, we have generated the first manually labeled validation data set of SO and non-SO events and used it to validate the performance of traditionally used SO detection algorithms. Furthermore, we have implemented a DTW-1NN together with machine and deep learning approaches which show an improved performance in terms of SO detection. While, prior to training, the outlined procedure is semi-automatic, requiring labeling of some events, the trained algorithms are fully automatic, allowing fast labeling of any number of recordings. We believe the current tool and developed algorithms are of significant importance for sleep researchers for two reasons: on the one hand, the tool and pre-filtering procedure allow for validation of existing and newly developed SO detection algorithms and on the other hand, our deep learning approach offers superior performance compared to that of traditional SO detection algorithms. We recommend using these algorithms in future work and, to that end, have made them publicly available in our tool *Sleepy* (Donle et al., 2022).

## Data availability statement

The original contributions presented in the study are included in the article/Supplementary material, further inquiries can be directed to the corresponding author.

## Ethics statement

The studies involving humans were approved by the Local Ethics Committee at Universitätsmedizin Greifswald. The studies were conducted in accordance with the local legislation and institutional requirements. The participants provided their written informed consent to participate in this study.

## Author contributions

CD: Conceptualization, Formal analysis, Methodology, Software, Visualization, Writing— original draft, Writing—review & editing. LD: Formal analysis, Software, Visualization, Writing—original draft, Writing—review & editing. CC: Conceptualization, Writing—review & editing. TG: Conceptualization, Writing—review & editing. LK: Data curation, Validation, Writing—review & editing. JL: Data curation, Validation, Writing—review & editing. AF: Funding acquisition, Supervision, Writing—review & editing. KO: Funding acquisition, Supervision, Writing—review & editing.

## References

[B1] Achermann P. Borbély A. (1997). Low-frequency (< 1hz) oscillations in the human sleep electroencephalogram. Neuroscience 81, 213–222. 10.1016/S0306-4522(97)00186-3 9300413

[B2] Amzica F. Steriade M. (1998). Electrophysiological correlates of sleep delta waves. Electroencephalogr. Clin. Neurophysiol. 107, 69–83. 10.1016/S0013-4694(98)00051-0 9751278

[B3] Bagnall A. Lines J. (2014). An experimental evaluation of nearest neighbour time series classification. arXiv. [Preprint]. 10.48550/arXiv.1406.4757

[B4] Bagnall A. Lines J. Hills J. Bostrom A. (2015). Time-series classification with cote: the collective of transformation-based ensembles. IEEE Trans. Knowl. Data Eng. 27, 2522–2535. 10.1109/TKDE.2015.2416723

[B5] Bersagliere A. Achermann P. (2010). Slow oscillations in human non-rapid eye movement sleep electroencephalogram: effects of increased sleep pressure. J. Sleep Res. 19(1p2), 228–237. 10.1111/j.1365-2869.2009.00775.x 19845847

[B6] Cakan C. Dimulescu C. Khakimova L. Obst D. Flöel A. Obermayer K. (2022). Spatiotemporal patterns of adaptation-induced slow oscillations in a whole-brain model of slow-wave sleep. Front. comput. Neurosci. 15, 800101. 10.3389/fncom.2021.800101 35095451 PMC8790481

[B7] Criswell E. (2010). Cram's Introduction to Surface Electromyography. Sudbury, MA: Jones and Bartlett Publishers.

[B8] Dijk D.-J. Duffy J. F. Czeisler C. A. (2000). Contribution of circadian physiology and sleep homeostasis to age-related changes in human sleep. Chronobiol. Int. 17, 285–311. 10.1081/CBI-100101049 10841208

[B9] Donle L. Cakan C. Dimulescu C. (2022). caglorithm/sleepy: Sleepy. 10.5281/zenodo.7115257

[B10] Dubé J. Lafortune M. Bedetti C. Bouchard M. Gagnon J. F. Doyon J.. (2015). Cortical thinning explains changes in sleep slow waves during adulthood. J. Neurosci. 35, 7795–7807. 10.1523/JNEUROSCI.3956-14.2015 25995467 PMC6795194

[B11] Edwards B. A. O'Driscoll D. M. Ali A. Jordan A. S. Trinder J. Malhotra A.. (2010). Aging and sleep: physiology and pathophysiology. Semin. Respir. Crit. Care Med. 31, 618–633. 10.1055/s-0030-1265902 20941662 PMC3500384

[B12] Esser S. K. Hill S. L. Tononi G. (2007). Sleep homeostasis and cortical synchronization: I. Modeling the effects of synaptic strength on sleep slow waves. Sleep 30, 1617–1630. 10.1093/sleep/30.12.1617 18246972 PMC2276134

[B13] Heib D. P. Hoedlmoser K. Anderer P. Zeitlhofer J. Gruber G. Klimesch W.. (2013). Slow oscillation amplitudes and up-state lengths relate to memory improvement. PLoS ONE 8, e82049. 10.1371/journal.pone.0082049 24324743 PMC3852994

[B14] Janocha K. Czarnecki W. M. (2017). On loss functions for deep neural networks in classification. Comput. Res. Repository, abs/1702.05659. 10.48550/arXiv.1702.05659

[B15] Jung T.-P. Humphries C. Lee T.-W. Makeig S. McKeown M. Iragui V.. (1997). Extended ICA removes artifacts from electroencephalographic recordings. Adv. Neural Inf. Process. Syst. 10, 894–900.

[B16] Jung T.-P. Makeig S. Westerfield M. Townsend J. Courchesne E. Sejnowski T. J.. (2000). Removal of eye activity artifacts from visual event-related potentials in normal and clinical subjects. Clin. Neurophysiol. 111, 1745–1758. 10.1016/S1388-2457(00)00386-2 11018488

[B17] Kandel I. Castelli M. (2020). The effect of batch size on the generalizability of the convolutional neural networks on a histopathology dataset. ICT Express 6, 312–315. 10.1016/j.icte.2020.04.010

[B18] Kate R. (2015). Using dynamic time warping distances as features for improved time series classification. Data Min. Knowl. Discov. 30, 283–312. 10.1007/s10618-015-0418-x

[B19] Keogh E. Ratanamahatana C. A. (2005). Exact indexing of dynamic time warping. Knowl. Inf. Syst. 7, 358–386. 10.1007/s10115-004-0154-9

[B20] Kingma D. P. Ba J. (2015). Adam: a method for stochastic optimization. Comput. Res. Repository, abs/1412.6980. 10.48550/arXiv.1412.6980

[B21] Ladenbauer J. Khakimova L. Malinowski R. Obst D. Tonnies E. Antonenko D.. (2021). Towards optimization of oscillatory stimulation during sleep. bioRxiv. 10.1101/2021.09.27.461932 35981956

[B22] Ladenbauer J. Ladenbauer J. Külzow N. de Boor R. Avramova E. Grittner U.. (2017). Promoting sleep oscillations and their functional coupling by transcranial stimulation enhances memory consolidation in mild cognitive impairment. J. Neurosci. 37, 7111–7124. 10.1523/JNEUROSCI.0260-17.2017 28637840 PMC6705731

[B23] Leissner P. Lindholm L.-E. Petersen I. (1970). Alpha amplitude dependence on skull thickness as measured by ultrasound technique. Electroencephalogr. Clin. Neurophysiol. 29, 392–399. 10.1016/0013-4694(70)90047-7 4097209

[B24] Makeig S. Jung T.-P. Bell A. J. Ghahremani D. Sejnowski T. J. (1997). Blind separation of auditory event-related brain responses into independent components. Proc. Nat. Acad. Sci. 94, 10979–10984. 10.1073/pnas.94.20.10979 9380745 PMC23551

[B25] Mander B. A. Marks S. M. Vogel J. W. Rao V. Lu B. Saletin J. M.. (2015). β-amyloid disrupts human NREM slow waves and related hippocampus-dependent memory consolidation. Nat. Neurosci. 18, 1051–1057. 10.1038/nn.4035 26030850 PMC4482795

[B26] Massimini M. Huber R. Ferrarelli F. Hill S. Tononi G. (2004). The sleep slow oscillation as a traveling wave. J. Neurosci. 24, 6862–6870. 10.1523/JNEUROSCI.1318-04.2004 15295020 PMC6729597

[B27] Mensen A. Riedner B. Tononi G. (2016). Optimizing detection and analysis of slow waves in sleep EEG. J. Neurosci. Methods 274, 1–12. 10.1016/j.jneumeth.2016.09.006 27663980

[B28] Mikutta C. Feige B. Maier J. G. Hertenstein E. Holz J. Riemann D.. (2019). Phase-amplitude coupling of sleep slow oscillatory and spindle activity correlates with overnight memory consolidation. J. Sleep Res. 28, e12835. 10.1111/jsr.12835 30848042

[B29] Mölle M. Eschenko O. Gais S. Sara S. J. Born J. (2009). The influence of learning on sleep slow oscillations and associated spindles and ripples in humans and rats. Eur. J. Neurosci. 29, 1071–1081. 10.1111/j.1460-9568.2009.06654.x 19245368

[B30] Muehlroth B. E. Sander M. C. Fandakova Y. Grandy T. H. Rasch B. Shing Y. L.. (2019). Precise slow oscillation-spindle coupling promotes memory consolidation in younger and older adults. Sci. Rep. 9, 1–15. 10.1038/s41598-018-36557-z 30760741 PMC6374430

[B31] Muehlroth B. E. Werkle-Bergner M. (2020). Understanding the interplay of sleep and aging: methodological challenges. Psychophysiology 57, e13523. 10.1111/psyp.13523 31930523

[B32] Ngo H.-V. V. Martinetz T. Born J. Mölle M. (2013). Auditory closed-loop stimulation of the sleep slow oscillation enhances memory. Neuron 78, 545–553. 10.1016/j.neuron.2013.03.006 23583623

[B33] Nolan H. Whelan R. Reilly R. B. (2010). Faster: fully automated statistical thresholding for eeg artifact rejection. J. Neurosci. Methods 192, 152–162. 10.1016/j.jneumeth.2010.07.015 20654646

[B34] NVIDIA Vingelmann P. Fitzek F. H. (2020). Cuda, release: 10.2.89.

[B35] Oostenveld R. Fries P. Maris E. Schoffelen J.-M. (2011). Fieldtrip: open source software for advanced analysis of meg, eeg, and invasive electrophysiological data. Comput. Intell. Neurosci. 2011, 1–9. 10.1155/2011/156869 21253357 PMC3021840

[B36] Perrin F. Pernier J. Bertrand O. Echallier J. F. (1989). Spherical splines for scalp potential and current density mapping. Electroencephalogr. Clin. Neurophysiol. 72, 184–187. 10.1016/0013-4694(89)90180-6 2464490

[B37] Petitjean F. Ketterlin A. Gançarski P. (2011). A global averaging method for dynamic time warping, with applications to clustering. Pattern Recognit. 44, 678–693. 10.1016/j.patcog.2010.09.013

[B38] Rasch B. Born J. (2013). About sleep's role in memory. Physiol. Rev. 93, 681–766. 10.1152/physrev.00032.2012 23589831 PMC3768102

[B39] Rechtschaffen A. (1968). A manual for standardized terminology, techniques and scoring system for sleep stages in human subjects. Brain Inform. Serv. 11422885 10.1046/j.1440-1819.2001.00810.x

[B40] Sakoe H. (1971). “Dynamic-programming approach to continuous speech recognition,” in 1971 Proc. the International Congress of Acoustics (Budapest).

[B41] Sakoe H. Chiba S. (1978). Dynamic programming algorithm optimization for spoken word recognition. IEEE Trans. Acoust. 26, 43–49. 10.1109/TASSP.1978.1163055

[B42] Segalowitz S. Davies P. L. (2004). Charting the maturation of the frontal lobe: an electrophysiological strategy. Brain Cogn. 55, 116–133. 10.1016/S0278-2626(03)00283-5 15134847

[B43] Shi X. Chen Z. Wang H. Yeung D. Wong W. Woo W.. (2015). Convolutional LSTM network: a machine learning approach for precipitation nowcasting. Comput. Res. Repository, abs/1506.04214.

[B44] Silva D. F. Batista G. E. (2016). “Speeding up all-pairwise dynamic time warping matrix calculation,” in Proceedings of the 2016 SIAM International Conference on Data Mining (SIAM), 837–845. 10.1137/1.9781611974348.94

[B45] Smith L. N. (2015). No more pesky learning rate guessing games. Comput. Res. Repository, abs/1506.01186.

[B46] Smith S. L. Kindermans P. Le Q. V. (2017). Don't decay the learning rate, increase the batch size. Comput. Res. Repository, abs/1711.00489. 10.48550/arXiv.1711.00489

[B47] Tan C. W. Webb G. I. Petitjean F. (2017). “Indexing and classifying gigabytes of time series under time warping,” in Proceedings of the 2017 SIAM International Conference on Data Mining (SIAM), 282–290. 10.1137/1.9781611974973.32

[B48] Tan C. W. Herrmann M. Forestier G. Webb G. I. Petitjean F. (2018). “Efficient search of the best warping window for dynamic time warping,” in Proceedings of the 2018 SIAM International Conference on Data Mining (SIAM), 225–233. 10.1137/1.9781611975321.26

[B49] Timofeev I. Grenier F. Bazhenov M. Sejnowski T. Steriade M. (2000). Origin of slow cortical oscillations in deafferented cortical slabs. Cereb. Cortex 10, 1185–1199. 10.1093/cercor/10.12.1185 11073868

[B50] Timofeev I. Schoch S. F. LeBourgeois M. K. Huber R. Riedner B. A. Kurth S. (2020). Spatio-temporal properties of sleep slow waves and implications for development. Curr. Opin. Physiol. 15, 172–182. 10.1016/j.cophys.2020.01.007 32455180 PMC7243595

[B51] Vyazovskiy V. V. Olcese U. Lazimy Y. M. Faraguna U. Esser S. K. Williams J. C.. (2009). Cortical firing and sleep homeostasis. Neuron 63, 865–878. 10.1016/j.neuron.2009.08.024 19778514 PMC2819325

