# Supplementary material for: Improving the detection of sleep slow oscillations in electroencephalographic data
Source: Front Neuroinform. 2024 Feb 5;18:1338886. doi: 10.3389/fninf.2024.1338886 (PMC10875054; doi:10.3389/fninf.2024.1338886)
Supplement: Supplementary file 1 [file Data_Sheet_1.pdf]

Supplementary Material

1 SUPPLEMENTARY TABLES AND FIGURES

1.1 Pre-filtering procedure

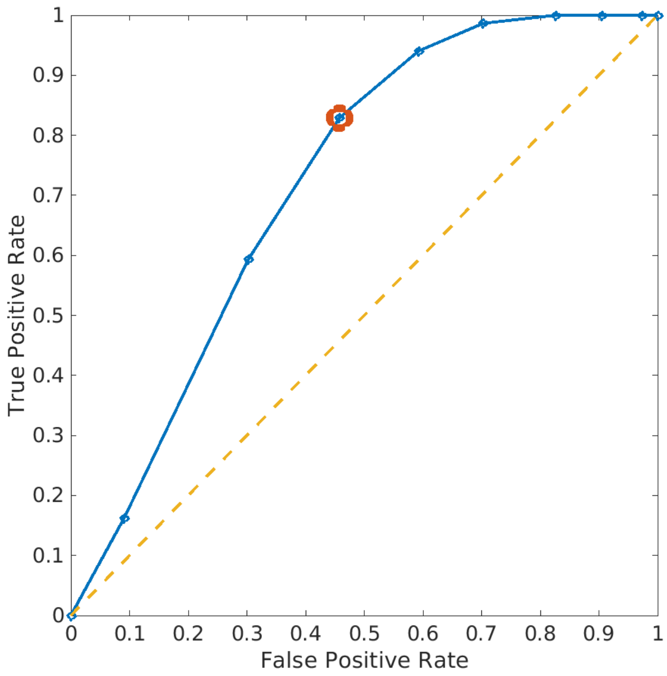

Figure S1: Example for determining the exclusion-inclusion criteria of the pre-filtering procedure showing the total SO duration criterion for one participant. The yellow dotted line indicates the random classifier, i.e. points on this line would indicate random guesses. The blue line shows the performance of the pre-filtering procedure. Each dot represents one value of the total SO duration criterion for this participant, as described in Section 2.2.1. The red circle marks the optimal criterion value. Lowering the criterion beyond this value would lead to a larger loss of true positive events compared to the elimination of false positive events.

Crit. / Part.	1 [s]	2 [s]	3 [s]	4 [s]	5 [#]	6 [a.u.]	7 [a.u.]
1	1.67	0.64	0.98	0.82	2	0.68	0.17
2	2.51	1.48	1.76	0.96	1	1.60	0.01
3	2.10	1.11	1.16	1.10	1	0.94	0.26
4	2.10	0.94	1.19	1.02	1	0.54	0.0
5	1.65	0.91	0.83	0.98	2	0.69	0.40
6	2.08	1.15	1.17	1.34	1	0.17	0.02
7	2.09	1.03	1.34	1.66	3	0.89	0.53
8	2.24	0.83	1.58	1.30	1	0.79	0.51
9	2.21	0.86	1.50	0.94	1	1.34	0.11
10	2.37	1.04	1.55	1.36	1	0.87	0.47

Table S1. Parameter values obtained from the pre-filtering procedure applied to each of the 10 participants. The seven criteria (top row) are the same as described in Section 2.2.1, as follows: 1 - maximum SO duration, 2 - duration from first positive-to-negative zero-crossing to the SO negative peak, 3 - duration from the negative to the positive SO peak, 4 - duration from the positive SO peak to the second positive-to-negative zero-crossing, 5 - number of positive SO peaks, 6 - scaling factor for negative-to-positive peak-to-peak amplitude, 7 - scaling factor for negative peak voltage.

Subject	% TP	% FP
1	84.04	14.01
2	83.92	5.00
3	86.74	8.49
4	83.47	22.76
5	79.67	2.80
6	80.69	53.05
7	92.84	5.30
8	88.42	20.41
9	83.76	4.03
10	95.20	5.06

Table S2. Percentages of remaining true (TP) and false positives (FP) for each participant's fully manually labelled channel after applying the pre-filtering procedure.

1.2 Data workflow

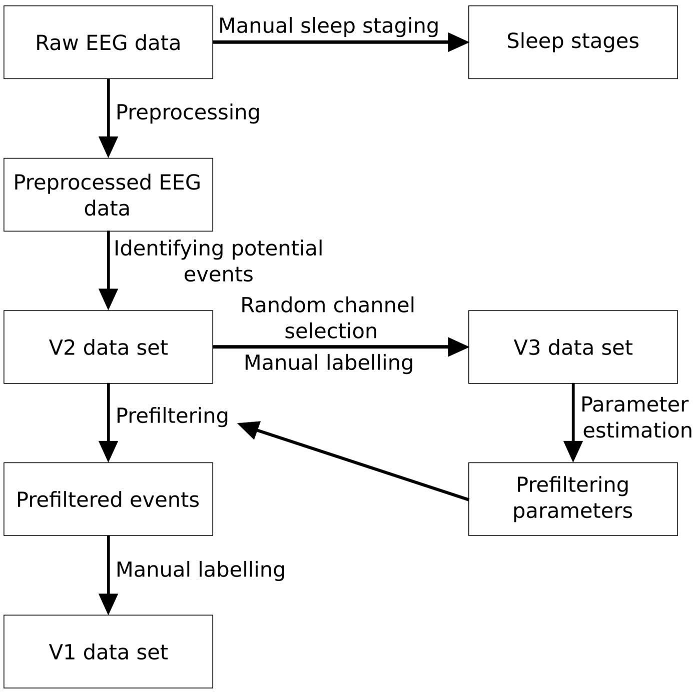

Figure S2: Workflow overview of all steps conducted for obtaining the three data sets detailed in Section 2.5.2. Manual sleep staging is detailed in Section 2.1.3. Data preprocessing was conducted using custom in-house FieldTrip scripts and is detailed in Section 2.1.4. All other steps were conducted using sleepy and are detailed in Section 2.2.

1.3 Descriptive SO statistics for the automatic detection algorithms

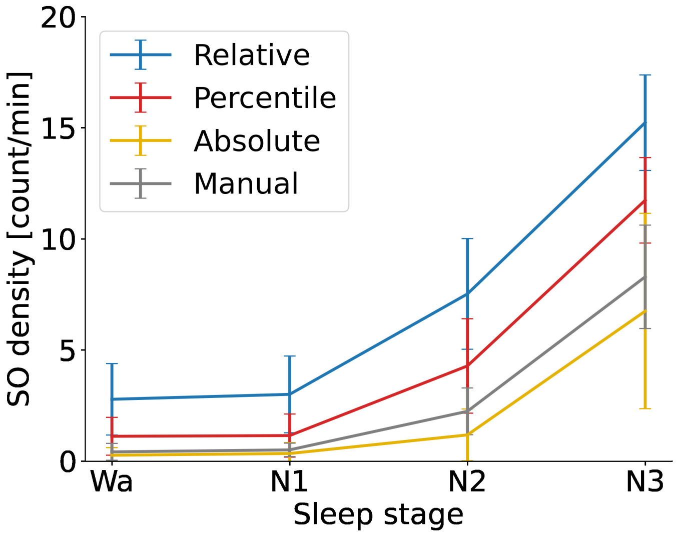

Figure S3: Average and standard deviation of the SO density (number of SOs per minute) across participants for each the three automatic detection algorithms applied to the preprocessed data as described in Section 2.3 and the manually labelled data set (V1) for each of the three sleep stages (N1 - N3) and the awake state (Wa).

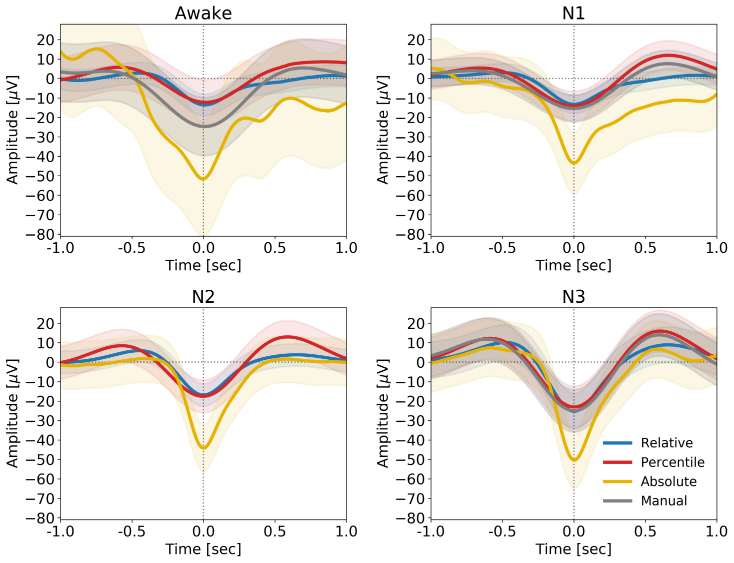

Figure S4: Average SO waveform across the three automatic detection algorithms and the manually labelled data set obtained according to the procedure described in Sections 2.2.1. and 2.2.2. for each of the three sleep stages (N1 - N3) and the awake state (Wa). The solid lines mark the average negative-to-positive peak-to-peak amplitude for each of the three automatic detection algorithms (blue - relative, red - percentile, yellow - absolute) and the manually labelled data (gray), while the shaded area represents the standard deviation around the mean. In each of the four cases (awake state and sleep stages N1 - N3), the absolute algorithm shows the highest amplitude, while the relative and the percentile algorithm more closely track the manually labelled data. The amplitude of the detected events is largest in sleep stage N3 compared to the other sleep stages for all three automatic algorithms and for the manually labelled data.

1.4 EEG features used by the Machine Learning approaches

Table S3 summarizes the features generated for each sequence identified as a possible SO event after manual labelling. The first nine features, obtained with a custom Python implementation, provide general properties of the sequence, such as the total length of the sequence in seconds from the first to the last zero crossing (start to end), the minimum, maximum, and through-to-peak amplitude of the sequence in μV, the ratio of the absolute values of minimum to maximum, the sequence lengths from the beginning to the middle zero-crossing (ZC) and from the middle zero-crossing (ZC) to the end, the number of local extrema determined by the zero crossings of the derivative, and the number of turning points determined by the zero crossings of the second derivative. The next three features are derived from a discrete wavelet transform (DWT) and correspond to the maximum value, the minimum value, and the associated ratio of the single-level detail coefficient of a DWT with a second-order Daubechies wavelet (db2). The Python pywt.dwt library was used for this purpose. The next two features are the frequency of the component with the highest discrete Short-Time Fourier Transform (STFT) magnitude of the signal and the corresponding time of the window, determined using the Python scipy.signal.stft library. The last three features contain the three frequencies corresponding to the three highest values of the spectral power obtained by Fourier transform, determined using the Python numpy.fft library.

Category	Feature
General properties of the sequence	Sequence length [s]
	Minimum of the sequence [μV]
	Maximum of the sequence [μV]
	Trough-to-peak amplitude [μV]
	Ratio of minimum to maximum
	Sequence length from start until middle ZC [s]
	Sequence length from middle ZC until end [s]
	Number of local extrema
	Number of turning points
Discrete Wavelet Transformation	Maximum of detail coefficient [μV]
	Minimum of detail coefficient[μV]
	The ratio of the previous two features
Short Time Fourier Transformation (STFT)	Time of maximal STFT level [s]
	Frequency of maximal STFT level [Hz]
Spectral Powers	Strongest frequency [Hz]
	2nd strongest frequency [Hz]
	3rd strongest frequency [Hz]

Table S3. Summary of the features used by the Machine Learning approaches

1.5 Hyperparameter Optimization for Deep Learning and resulting Architectures

In Table S4 the hyperparameters are shown, which have been set equally for all Deep Learning models.

Hyperparameter	Value
Learning rate	5e-4
Epochs	1,000
Batch Size	64
Loss Function	Binary Cross-entropy
Optimizer	Adam

Table S4. General hyperparameter values

In Figure S5 the architectures resulting from the structural hyperparameter optimization process for each type of Deep Learning model is shown.

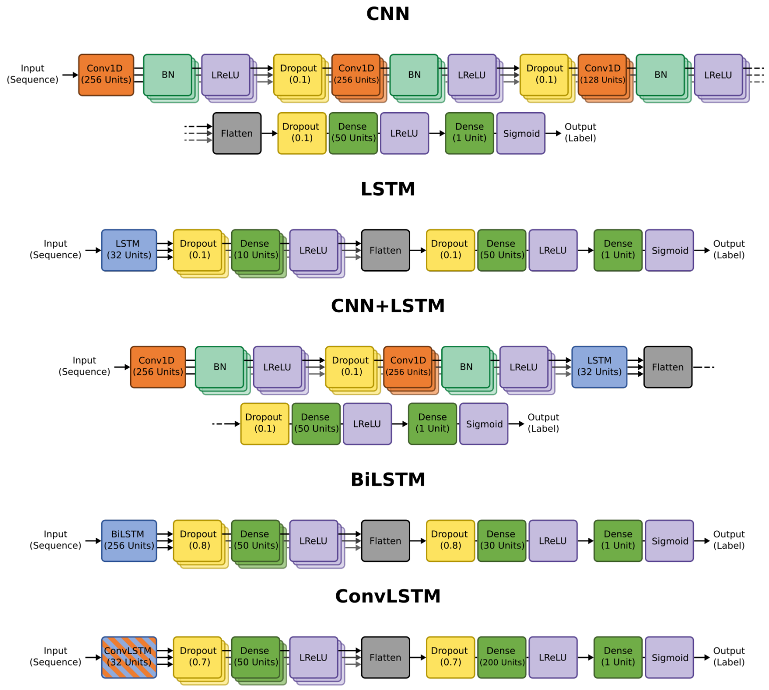

Figure S5: Hyperparameter optimized architecture for each type of Deep Learning model. LReLU stands for Leaky Rectifier Linear Unit, BN for Batch Normalization.

1.6 Multi-participant vs. Single-participant Models

Figure S6 shows the performance on the test set as a function of training set size for the best performing Machine Learning (Random Forest with RFE) and Deep Learning (BiLSTM) approaches for multi-participant and single-participant models. For each subject, the single-participant models were trained on sequences from a single participant only and had to recognize SOs during test only for that participant. Multi-participant models were trained using data from all participants and had to recognize SOs during test across the participants. Figure S6 shows that single-participant models do not improve performance. This could be due to the higher absolute amount of training data for the multi-participant model. While the multi-participant model has a larger absolute amount of training data across the participants, but the same amount in regard to a single participant, a single participant-based model has a smaller amount of absolute data from coming only from a single participant, but the accumulation of all participant-based models has the same amount of data as the multi-participant model. The Machine Learning and Deep Learning approaches benefit greatly from more data, so it is reasonable that the multi-participant model has higher predictive power.

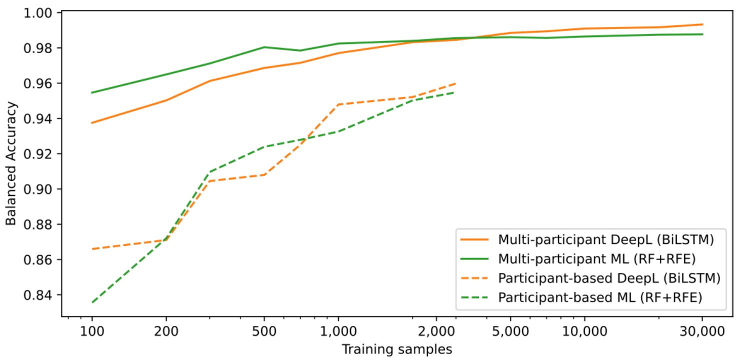

Figure S6: Balanced accuracy as a function of training set size for single-participant (dashed lines) and multi-participant (solid lines) models. Line color denotes ML (green) and DeepL (orange) approaches.

1.7 Effect of the number of training data on classifier validation performance

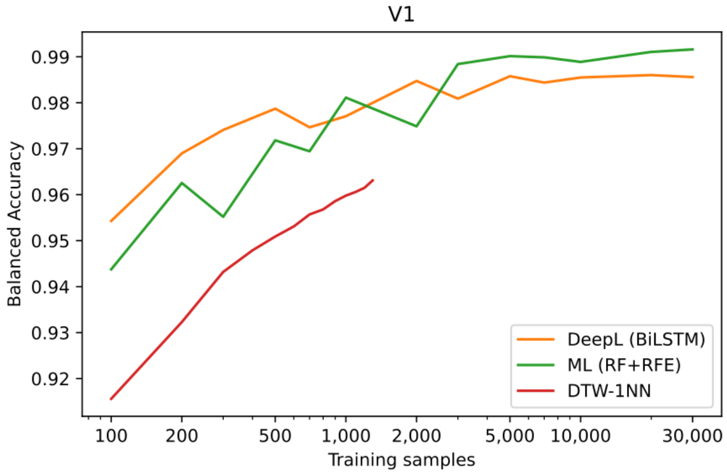

Figure S7: Results on the Validation data set V1 in terms of balanced accuracy for different methods and for different quantities of training samples.

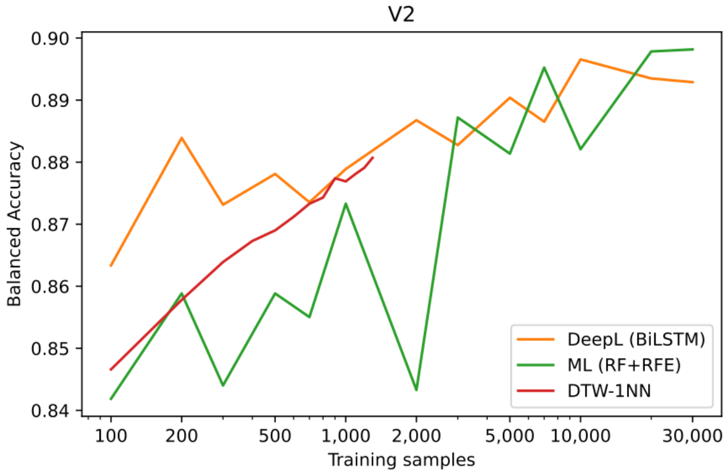

Figure S8: Results on the Validation data set V2 in terms of balanced accuracy for different methods and for different quantities of training samples.

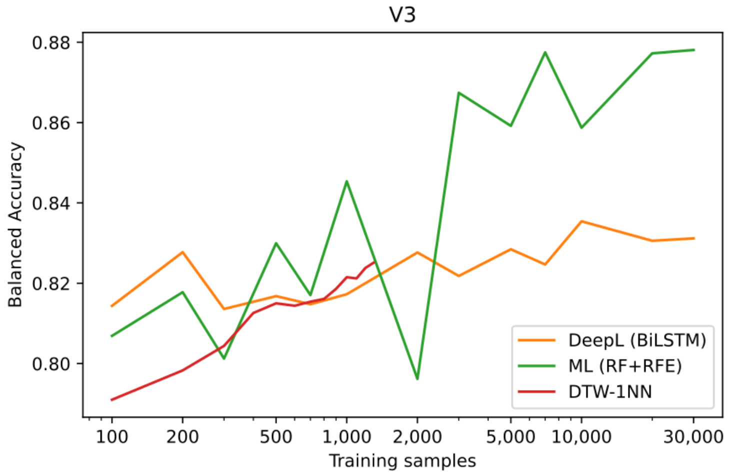

Figure S9: Results on the Validation data set V3 in terms of balanced accuracy for different methods and for different quantities of training samples.
